# Patients With IBD Receiving Methotrexate Are at Higher Risk of Liver Injury Compared With Patients With Non-IBD Diseases: A Meta-Analysis and Systematic Review

**DOI:** 10.3389/fmed.2021.774824

**Published:** 2021-11-22

**Authors:** Yang Wang, Yimin Li, Yun Liu, Yifan Zhang, Ziliang Ke, Yu Zhang, Yulan Liu

**Affiliations:** ^1^Department of Gastroenterology, Peking University People's Hospital, Beijing, China; ^2^Clinical Center of Immune-Mediated Digestive Diseases, Peking University People's Hospital, Beijing, China; ^3^Beijing Key Laboratory for Rheumatism Mechanism and Immune Diagnosis (BZ0135), Department of Rheumatology and Immunology, Peking University People's Hospital, Beijing, China

**Keywords:** inflammatory bowel disease, methotrexate, liver injury, hepatotoxicity, meta-analysis

## Abstract

**Background:** Methotrexate is well-known in treating inflammatory bowel disease (IBD), rheumatoid arthritis (RA), psoriasis (Ps), and psoriatic arthritis (PsA). Several reports have indicated a higher incidence of methotrexate (MTX)-related liver adverse events in patients with IBD. We aim to investigate the risk of liver injury in patients with IBD and those with non-IBD diseases treated with MTX.

**Methods:** We searched PubMed, Embase, and the Cochrane Library for articles that reported liver adverse events in patients with IBD, RA, and Ps/PsA, receiving MTX therapy. Additional articles were obtained by screening the references of recent meta-analysis and reviews. Raw data from included articles were pooled to calculate the cumulative incidence of total liver injury (TLI), MTX discontinuation (MTX-D), and liver fibrosis (LF). RR (relative risk) was calculated to compare the difference between patients with IBD and those with non-IBD diseases.

**Results:** A total of 326 articles with 128,876 patients were included. The patients with IBD had higher incidence of TLI [11.2 vs. 9.2%; relative risk (RR) = 1.22; *P* = 0.224] and MTX-D (2.6 vs. 1.8%; RR, 1.48; *P* = 0.089) than patients with non-IBD diseases. Due to the publication bias, trim-and-fill was performed. Afterwards, the patients with IBD showed significantly higher risk of TLI (11.2 vs. 3%; RR = 3.76; *p* < 0.001), MTX-D (3.3 vs. 0.7%; RR = 5; *p* < 0.001) and LF (3.1 vs. 0.1%; RR = 38.62; *P* = 0.001) compared with patients with non-IBD diseases.

**Conclusion:** IBD is associated with a higher risk of MTX-related liver injury. The mechanism of MTX-induced hepatotoxicity might be different in IBD and non-IBD diseases, and needs to be verified in future research.

## Introduction

Methotrexate is a folate antagonist that competitively inhibits dihydrofolate reductase, an enzyme for the synthesis of purines and pyrimidines, thus possessing potent anti-proliferative and anti-inflammatory effects on cells (1). Several clinical trials have shown the efficacy of MTX for inducing and maintaining remission in patients with inflammatory bowel disease (IBD) (2, 3). MTX is also commonly used as the mainstay of long-term immunosuppression therapy in rheumatoid arthritis (RA) (4), psoriasis (Ps) (5), and psoriatic arthritis (PsA) (6). However, due to the drug toxicity, long-term MTX therapy is associated with some side effects, most commonly including the gastrointestinal tract symptoms, such as nausea and diarrhea, liver injury, myelosuppression, and interstitial pneumonitis (7). MTX toxicity is one of the major reasons for discontinuation of treatment. The intra-hepatocellular accumulation of polyglutamated metabolite of MTX might be related with liver toxic effects, yet the precise mechanism of MTX-induced hepatotoxicity remains poorly understood.

Notably, the risk of MTX-related adverse events, especially liver toxicity, might vary between patients with different diseases. Previous reports have indicated a high incidence of MTX-induced liver injury in patients with IBD. Feagan et al. reported that 17.5% (11 out of 63) patients with IBD receiving MTX treatment had abnormal serum aminotransferase (8). In a retrospective research by Fournier et al., 24% (*n* = 16 of 67), patients with IBD developed abnormal liver function during MTX therapy (9). In another study by González-Lama et al., investigating the MTX-related liver abnormality in IBD, at least 20.8% (16 out of 77) patients presented with liver function abnormalities or significant liver fibrosis (10).

In contrast, the risk of liver adverse effects has been considered to be low in patients with non-IBD diseases (11). In a prospective study by Lie et al., a total of 430 patients with PsA and 1,218 with RA were investigated, and the overall reported incidences of liver enzyme elevations were both <10% (12). Recently, Gelfand et al. conducted a population-based study, which included 28,030, 5,687, and 6,520 patients with RA, Ps, and PsA, respectively, and reported the incidence rates of liver disease outcomes not exceeding 10 incidence rates per 1,000 person-years (13). Karlsson Sundbaum et al. performed 6,288 alanine aminotransferase (ALT) tests during a long-term follow-up of 213 RA patients receiving MTX and found only 7% of ALT tests were over the upper limit of normal (ULN) (14).

However, the treatment regimen can be diverse between IBD and non-IBD diseases in terms of the duration, dose of MTX, as well as folic acid supplementation. The risk of MTX-related liver injury in IBD needs to be further clarified. In this meta-analysis, we aim to thoroughly elucidate the risk of liver injury in patients with IBD and non-IBD diseases receiving MTX.

## Methods

### Search Strategy and Selection Criteria

We searched PubMed, Embase, and the Cochrane Library using the keywords of “IBD,” “inflammatory bowel,” “Crohn,” “ulcerative,” and “colitis” for IBD; “rheumatoid” and “rheumatoid arthritis” for RA; “psoriasis” for Ps; “psoriatic arthritis” for PsA; “liver,” “enzyme,” “fibrosis,” “hepatic,” “ALT,” “AST,” “aminopherase,” “aminotransferase,” and “transaminase” for liver injury; “methotrexate” and “MTX” for MTX. The citations of included articles, relevant reviews, recent meta-analysis were also screened manually for additional articles missed in the above database search. Search records were managed with EndNote (version X7) for articles screening. Preferred Reporting Items for Systematic Reviews and Meta-Analyses (PRISMA) guidelines were followed. The protocol of this meta-analysis is registered with the International Prospective Register of Systematic Reviews (PROSPERO, registration ID: CRD42021246278).

The eligibility of articles was determined by two authors (YW and YML) independently, and divergences of results were resolved *via* discussion with another author (YL). Selection and exclusion criteria: articles reporting the incidence of an abnormal liver function test or liver fibrosis were considered eligible for inclusion; while the articles reporting the combination of MTX with other medications, which had distinct hepatotoxicity, were excluded.

At title and abstract screening, the following articles were excluded: duplicates, reviews, meta-analysis, case reports, letters, clinical studies with no more than 20 patients, basic studies, non-relevant articles, and those written in languages other than English. Then full-text review was performed according to the selection criteria, and articles without needed data were excluded.

### Data Extraction and Definitions

Data were extracted by two authors (YW and YML) independently. Dissonance was resolved with another author (ZLK) by discussion and consensus. The following data were extracted: author, year of publication, study region, number of patients, age of population, the dose and the duration of MTX treatment, the concomitant medication, including steroid and folic acid, incidence of total liver injury (TLI), MTX discontinuation (MTX-D), and liver fibrosis (LF).

Since the description of MTX usage was different in the included articles, three criteria for the dosages of MTX were considered as “high dose”: (1) weekly dose > 15 mg/week, or (2) cumulative dose > 1,500 mg, or (3) > the 12.5 mg/m^2^ body surface area. Otherwise, the doses were considered as low. These cut-off values were determined according to the data of included articles. For example, the mean dose of MTX was <15 mg/week in most of the included articles reporting the efficacy of “low-dose” MTX; thus, 15 mg/week was chosen as the cut-off value for weekly dose.

In the meta-analysis, most of the included articles (72%) considered > 1 ULN as abnormal liver function, while the minority (28%) considered > 2 ULN or > 3 ULN as abnormality. Therefore, we investigated the incidence of total liver injury (TLI) by using > 1ULN as the standard. If liver enzymes were not reported, liver fibrosis was used instead. MTX-D was defined as the withdraw of MTX due to liver adverse events such as abnormal liver function or liver fibrosis. LF was diagnosed by liver biopsy (Roenigk grade > = III) or non-invasive methods (such as transient elastography, *F* > = 3).

### Data Synthesis and Statistical Analysis

Due to substantial trial heterogeneity, a random effects analysis was performed on the cumulative incidence rates by using the package of *meta* in R version 4.0.2 (http://www.r-project.org/). The results are presented as the pooled estimate with 95% CIs. The heterogeneity was detected by the *Q* and *I*^2^ statistics. RR was calculated by comparing the estimate in patients with IBD with that in those with non-IBD. To explore the potential sources of heterogeneity, subgroup analysis was performed according to the characteristics of included articles such as publication date (before 2000 or after 2000), the region of study, type of study design (prospective or retrospective), type of population (children or adult), sample size (< = 100 or > 100), dose of MTX (low or high), duration of MTX treatment (short term or long term), usage of steroid, usage of folic acid, and the combination of MTX dose, duration, and usage of steroid/folic acid. Analysis was not performed in subgroups with the number of articles <5. A sensitivity analysis was also performed by omitting articles with a low Newcastle-Ottawa Scale (NOS) score.

### Assessment of Quality of Studies and Publication Bias

Newcastle-Ottawa Scale was calculated to assess the quality of included articles. To investigate the potential reporting bias, Funnel-plot, and Egger's test were performed. *P* < 0.05 for Egger's test was considered significant publication bias. If publication bias was indicated, trim-and-fill was used for adjusting relative risk (RR).

The original dataset used for analysis is available in the Supplementary Materials.

## Results

### Search Results and Study Characteristics

**Figure 1:**
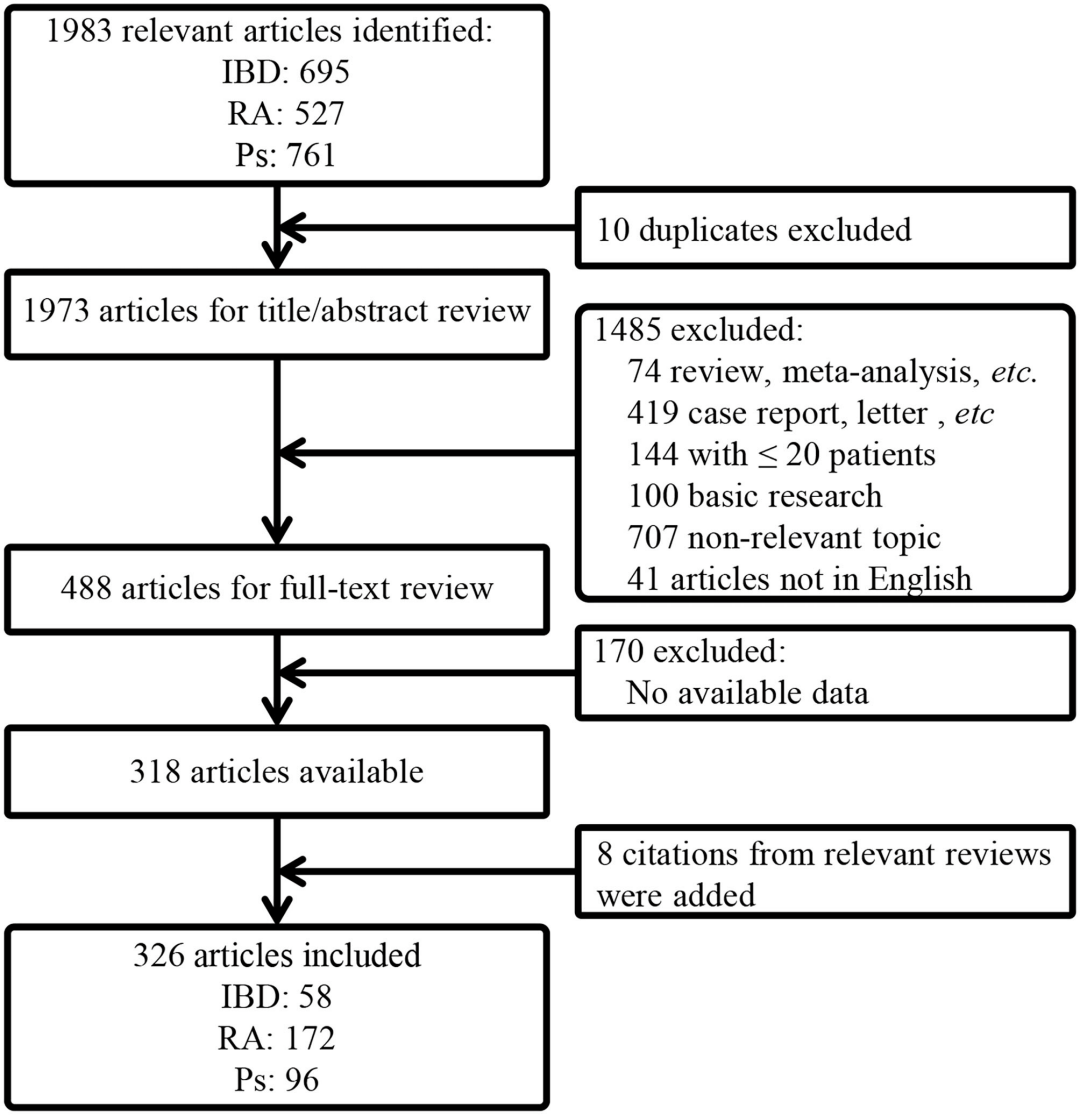
A flow chart of article selection. IBD, inflammatory bowel disease; RA, rheumatoid arthritis; Ps, psoriasis; PsA, psoriatic arthritis.

The article selection process is depicted in Figure 1. A total of 1,983 articles were initially identified. After removal of duplicates and screening *via* titles/abstracts and full-text review, a total of 326 articles, including 128,876 patients, were finally included (the reference list is available in Supplementary References).

The characteristics of the included articles are shown in Table 1. There were significant differences between the characteristics of IBD and non-IBD studies in terms of the region of study, study design, type of population, sample size, the administration pathway of MTX (intramuscularly/orally/subcutaneously/intravenously/combination), the dose of MTX, and the usage of steroid (*p* < 0.05 for all). Most of the included articles (*n* = 309) investigated adult population while the others (*n* = 17) studied children population. A higher proportion of articles investigating children population were observed in IBD (19 vs. 2.3%, *p* < 0.05). Notably, none of the IBD studies had a very long term of MTX duration; while quite a few non-IBD studies had a very long term more than 4 years. Given that longer term treatment of MTX is associated with a significantly elevated probability of hepatic toxicity, we excluded the 29 non-IBD articles with very long-term MTX treatment in the following comparison between IBD and non-IBD.

**Table 1:** Characteristics of studies included.

		**Non-IBD diseases**			
**Characteristics**	**IBD**	**Total (RA+Ps/PsA)**	**RA**	**Ps/PsA**	**Difference between 3 diseases (*P*-value)**
Total No. of articles	58	268	172	96	–
Total No. of patients	4,194	124,682	95,849	28,833	–
Male/female	2,032/2,162	63,252/61,430	48,925/46,924	14,327/14,506	–
**Year of publication (No. of articles)**					0.175
Before 2000	10	52	39	13	
After 2000	48	216	133	83	
**Region of study (No. of articles)**					<0.001
Europe	35	107	58	49	
North America	17	66	52	14	
Asia	4	88	57	31	
Oceania	2	3	3	0	
Africa	0	3	1	2	
South America	0	1	1	0	
**Study design (No. of articles)**					<0.001
Prospective	14	159	103	56	
Retrospective	44	109	69	40	
**Type of population (No. of articles)**					<0.001
Adult	47	262	169	93	
Children	11	6	3	3	
**Sample size (No. of articles)**					<0.001
≤ 100	46	114	53	61	
>100	12	154	119	35	
**Administration pathway of MTX**					<0.001
Intramuscularly	11	8	4	4	
Orally	10	117	69	48	
Subcutaneously	14	5	1	4	
Intravenously	1	0	0	0	
Combination	18	27	17	10	
**^a^** **Dose of MTX (No. of articles)**					<0.001
Low dose	19	160	103	57	
High dose	38	92	60	32	
**Mean/median duration of MTX treatment (No. of articles)**					0.067
Short term (≤ 2 years)	46	191	120	71	
Long term (2–4 years)	10	39	29	10	
Very long term (>4 years)	0	29	19	10	
**^b^** **Usage of steroid (No. of articles)**					<0.001
No	8	54	44	10	
Yes	40	67	63	4	
**^c^** **Usage of folic acid (No. of articles)**					0.631
No	6	21	16	5	
Yes	33	122	80	42	
**NOS score (No. of articles)**					0.803
5	2	15	11	4	
6	41	185	118	67	
7	1	3	3	0	
8	8	49	30	19	
9	6	16	10	6	

a *For the dose of MTX, low dose is defined as mean/median weekly dose ≤ 15 mg or 12.5 mg/m^2^, and cumulative dose ≤ 1.5 g; high dose is defined otherwise*.

b *For the usage of steroid, <50% patients receiving steroid is considered as No; otherwise Yes*.

c *For the usage of steroid, <50% patients receiving folic acid is considered as No; otherwise Yes*.

*IBD, inflammatory bowel disease; RA, rheumatoid arthritis; Ps, psoriasis; PsA, psoriatic arthritis; MTX, methotrexate; NOS, Newcastle-Ottawa Scale*.

Approximately, 25% of the articles were considered high quality with NOS score > 6. Since clinical trials comparing the incidence of MTX-related adverse events between IBD and non-IBD are scarce, we included single-arm research in this meta-analysis. Therefore, most of the included studies had a NOS score no more than 6.

### Risk of Total Liver Injury, MTX Discontinuation, and Liver Fibrosis in Patients With IBD and Patients With Non-IBD Diseases

As shown in Table 2, the cumulative incidence of TLI was 11.2% (95% CI, 8.1–14.7%) for IBD (Supplementary Figure S1), and 9.2% (95% CI, 8.1–10.3%) for non-IBD (Supplementary Figure S2). Although the incidence of TLI in IBD was higher than that in non-IBD (RR = 1.22; 95% CI, 0.88–1.68), the difference was not significant (*P* = 0.224). The comparison was further performed in different subgroups. In most of the subgroups (18 out of 22), there was a higher risk of TLI in patients with IBD (RR > 1). Notably, in the subgroups of prospective studies, usage of folic acid and folic acid + high dose/long term, the risk of TLI was significantly higher in patients with IBD (RR > 1, *p* < 0.05). While, in four subgroups, the difference was close to statistically significant (0.05 < *p* < 0.1).

**Table 2:** Incidence of total liver injury (TLI) in IBD and non-IBD patients receiving MTX.

	**IBD**			**Non-IBD**					
**Groups**	**No. of articles**	**Cumulative incidence**	**95% CI**	**No. of articles**	**Cumulative incidence**	**95% CI**	**RR**	**95% CI of RR**	***P* of RR**
Total	54	11.2%	8.1–14.7%	235	9.2%	8.1–10.3%	1.22	0.88–1.68	0.224
Subgroup: Year of publication, before 2000	6	16.4%	11–22.6%	42	18.6%	12.5–25.5%	0.88	0.53–1.46	0.634
Subgroup: Year of publication, after 2000	48	10.7%	7.5–14.4%	193	7.7%	6.7–8.7%	1.39	0.98–1.98	0.064
Subgroup: Region of study, North America	14	15%	5.7–27.4%	60	10.2%	8–12.6%	1.48	0.65–3.34	0.351
Subgroup: Region of study, Europe	35	9.5%	7.5–11.8%	93	8.4%	7–9.9%	1.14	0.86–1.5	0.373
Subgroup: Study design, prospective	10	14.3%	10.3–18.8%	149	8.9%	7.4–10.4%	1.62	1.14–2.29	0.007
Subgroup: Study design, retrospective	44	10.7%	7.2–14.6%	86	9.7%	8.2–11.4%	1.1	0.74–1.62	0.638
Subgroup: Children	11	12.3%	4.7–22.3%	6	11.2%	1.3–27.3%	1.1	0.2–6.12	0.916
Subgroup: Adult	43	10.9%	7.7–14.5%	229	9.1%	8.1–10.3%	1.19	0.85–1.67	0.317
Subgroup: Sample size, ≤ 100	42	12.2%	8.2–16.8%	99	10.3%	7.5–13.5%	1.18	0.74–1.88	0.486
Subgroup: Sample size, >100	12	8.5%	4.2–14.1%	136	8.7%	7.5–9.9%	0.99	0.53–1.84	0.965
Subgroup: Administration of MTX, intramuscularly	9	9.6%	5–15.3%	8	8.2%	2.8–15.7%	1.17	0.42–3.27	0.76
Subgroup: Administration of MTX, orally	9	10.4%	3.5–20.1%	116	9.1%	8.4–12%	1.14	0.4–2.43	0.97
Subgroup: Administration of MTX, subcutaneously	13	10.4%	5.5–16.5%	5	10.2%	0.3–29.8%	1.03	0.1–11.9	0.98
Subgroup: Administration of MTX, combination	18	13.3%	6.6–21.6%	27	10%	6.9–13.7%	1.32	0.66–2.63	0.43
Subgroup: Dose of MTX, low	17	10.1%	5.7–15.6%	153	9.2%	7.6–10.8%	1.1	0.65–1.88	0.721
Subgroup: Dose of MTX, high	36	12%	7.7–17%	66	8.2%	6.6–10%	1.45	0.93–2.27	0.098
Subgroup: Duration of MTX, short term	42	10.5%	7–14.6%	190	7.7%	6.6–8.8%	1.37	0.92–2.04	0.121
Subgroup: Duration of MTX, long term	10	16.1%	7.7–26.5%	38	17.7%	13.1–22.8%	0.91	0.46–1.78	0.778
Subgroup: Usage of steroid, Yes	37	11.4%	8.3–15%	56	7.9%	5.7–10.5%	1.44	0.94–2.22	0.094
Subgroup: Usage of folic acid, Yes	33	12.1%	7.9–17.1%	113	7.5%	6.2–8.9%	1.61	1.05–2.47	0.03
Subgroup: Steroid + low dose + short term	11	11.6%	4.6–20.9%	31	5.3%	3.3–7.6%	2.2	0.93–5.22	0.073
Subgroup: Steroid + high dose/long term	26	11.1%	8.1–14.5%	22	11.4%	6.8–16.8%	0.98	0.57–1.68	0.938
Subgroup: Folic acid + low dose + short term	8	8.4%	4.5–13.3%	56	6.7%	5.2–8.4%	1.25	0.69–2.27	0.456
Subgroup: Folic acid + high dose/long term	25	13.3%	7.8–20%	53	7.8%	5.9–9.8%	1.72	1–2.94	0.048
Subgroup: Steroid/folic acid + low dose/short term	12	11%	4.6–19.4%	71	6.5%	5.1–8.1%	1.68	0.79–3.57	0.18
Subgroup: Steroid/folic acid + high dose/long term	34	12.2%	8–17.1%	66	9.5%	7.5–11.8%	1.28	0.82–2	0.276

*IBD, inflammatory bowel disease; CI, confidence interval; RR, relative risk; MTX, methotrexate*.

It has been reported that the incidence of MTX-toxicity is associated with a variety of factors, including the dose and the duration of MTX, the concomitant medication, such as steroid and folic acid. When these factors were restricted in the subgroups of steroid/folic acid + low-dose/short-term and steroid/folic acid + high dose/long term, the incidence of TLI was still higher in IBD than that in non-IBD [RR = 1.68 (95% CI, 0.79–3.57; *P* = 0.18) and 1.28 (95% CI, 0.82–2; *P* = 0.276), respectively]. The results were consistent in subgroups of different administration pathways of MTX (RR > 1 for all four subgroups). The subgroup of intravenously administered MTX was not evaluated due to limited number of articles (*n* = 1).

As shown in Table 3, the cumulative incidence of MTX-D was 2.6% (95% CI, 1.8–3.6%) in IBD (Supplementary Figure S3) and 1.8% (95% CI, 1.3–2.3%) in non-IBD (Supplementary Figure S4). The difference was close to significant (RR = 1.48; 95% CI, 0.94–2.33; *P* = 0.089). IBD also had a higher risk of MTX-D in most of the subgroups (22 out of 25; RR > 1). The difference was significant (*p* < 0.05) in five subgroups and close to significant (0.05 < *p* < 0.1) in four subgroups. When factors, such as the administration pathway of MTX, concomitant usage of steroid/folic acid, and dose/duration of MTX, were restricted, patients with IBD were still at higher risk of MTX-D (RR > 1).

**Table 3:** Incidence of MTX discontinuation (MTX-D) due to liver injury in IBD and non-IBD patients.

	**IBD**			**Non-IBD**					
**Groups**	**No. of articles**	**Cumulative incidence**	**95% CI**	**No. of articles**	**Cumulative incidence**	**95% CI**	**RR**	**95% CI of RR**	***P* of RR**
Total	39	2.6%	1.8- 3.6%	139	1.8%	1.3–2.3%	1.48	0.94–2.33	0.089
Subgroup: Year of publication, before 2000	9	2.8%	1.1–5.1%	30	3.2%	1.8–5%	0.86	0.34–2.21	0.76
Subgroup: Year of publication, after 2000	30	2.7%	1.7–3.8%	109	1.5%	1.1–2%	1.76	1.05–2.94	0.031
Subgroup: Region of study, North America	13	3.1%	1.7–4.6%	41	1.2%	0.5–2.1%	2.52	1.09–5.81	0.03
Subgroup: Region of study, Europe	21	2.7%	1.5–4.2%	57	3.2%	2.2–4.3%	0.85	0.46–1.58	0.609
Subgroup: Study design, prospective	10	2.1%	0.7–4.2%	95	1.5%	1–2.1%	1.39	0.51–3.75	0.516
Subgroup: Study design, retrospective	29	2.8%	1.8–3.9%	44	2.3%	1.4–3.5%	1.2	0.66–2.2	0.548
Subgroup: Children	10	2.7%	1.6–4.1%	5	2.1%	0–6.8%	1.29	0.05–34.85	0.881
Subgroup: Adult	29	2.6%	1.5–3.9%	134	1.8%	1.3–2.3%	1.48	0.86–2.56	0.159
Subgroup: Sample size, ≤ 100	35	2.5%	1.5–3.7%	66	2.4%	1.3–3.9%	1.04	0.52–2.1	0.911
Subgroup: Sample size, >100	4	NA^a^	NA	73	1.6%	1.1–2.1%	NA	NA	NA
Subgroup: Administration of MTX, intramuscularly	9	1.9%	0.4–4%	5	1.1%	0.2–2.5%	1.67	0.31–9	0.55
Subgroup: Administration of MTX, orally	8	2.8%	1.6–4.2%	68	2.5%	1.7–3.6%	1.08	0.58–2.03	0.8
Subgroup: Administration of MTX, subcutaneously	8	1.9%	0.6–3.7%	4	NA	NA	NA	NA	NA
Subgroup: Administration of MTX, combination	11	4.1%	1.9–6.9%	14	1.5%	0.4–3.2%	2.68	0.76–9.45	0.13
Subgroup: Dose of MTX, low	13	3.1%	1.8–4.7%	95	1.7%	1.1–2.3%	1.83	0.99–3.36	0.052
Subgroup: Dose of MTX, high	26	2.4%	1.3–3.6%	38	1.5%	0.8–2.4%	1.56	0.75–3.25	0.231
Subgroup: Duration of MTX, short term	34	2.5%	1.7–3.4%	115	1.8%	1.3–2.4%	1.38	0.86–2.24	0.186
Subgroup: Duration of MTX, long term	5	4.1%	0.5–9.9%	22	1.4%	0.5–2.6%	2.89	0.55–15.23	0.21
Subgroup: Usage of steroid, Yes	30	2.7%	1.8–3.8%	34	0.7%	0.2–1.2%	4.09	1.66–10.08	0.002
Subgroup: Usage of folic acid, Yes	21	3%	1.9–4.2%	64	1.9%	1.3–2.7%	1.53	0.89–2.65	0.127
Subgroup: Steroid + low dose + short term	9	3.3%	1.8–5.2%	21	0.8%	0.2–1.7%	4.24	1.12–16.13	0.034
Subgroup: Steroid + high dose/long term	21	2.5%	1.4–3.9%	11	0.5%	0–1.3%	5.16	0.74–36.14	0.098
Subgroup: Folic acid + low dose + short term	6	3.9%	1.9–6.5%	32	2.3%	1.3–3.6%	1.7	0.77–3.75	0.19
Subgroup: Folic acid + high dose/long term	15	2.6%	1.4–4.1%	30	1.2%	0.6–2%	2.12	0.95–4.75	0.067
Subgroup: Steroid/folic acid + low dose/short term	10	3.3%	1.9–5%	44	1.8%	1–2.7%	1.84	0.91–3.69	0.087
Subgroup: Steroid/folic acid + high dose/long term	24	2.7%	1.6–4%	38	1.1%	0.6–1.7%	2.52	1.24–5.11	0.01

a *NA indicates that analysis is not performed due to limited number of articles (<5)*.

*MTX, methotrexate; IBD, inflammatory bowel disease; CI, confidence interval; RR, relative risk*.

The cumulative incidence of LF (Table 4) was similar between IBD (Supplementary Figure S5) and non-IBD (Supplementary Figure S6) (3.6 vs. 3.7%; RR = 0.96; *P* = 0.933). However, the number of IBD studies reporting LF was quite limited (*n* = 9). In most of the subgroups, data analysis was invalid due to limited number of articles.

**Table 4:** Incidence of liver fibrosis (LF) in IBD and non-IBD patients receiving MTX.

	**IBD**			**Non-IBD**					
**Groups**	**No. of articles**	**Cumulative incidence**	**95% CI**	**No. of articles**	**Cumulative incidence**	**95% CI**	**RR**	**95% CI of RR**	***P* of RR**
Total	9	3.6%	1.3–6.7%	32	3.7%	2.5–5.2%	0.96	0.39–2.35	0.933
Subgroup: Year of publication, before 2000	2	NA^a^	NA	20	6.3%	2.9–10.5%	NA	NA	NA
Subgroup: Year of publication, after 2000	7	3.5%	0.9–7.3%	12	2%	1–3.2%	1.75	0.38–8.05	0.473
Subgroup: Region of study, North America	2	NA	NA	17	4.8%	2.2–8.3%	NA	NA	NA
Subgroup: Region of study, Europe	7	5.1%	2.9–7.8%	10	1.4%	0.2–3.2%	3.71	0.85–16.15	0.081
Subgroup: Study design, prospective	4	NA	NA	15	3.6%	1–7.3 %	NA	NA	NA
Subgroup: Study design, retrospective	5	3.8%	0.6–8.8%	17	4.3%	2.7–6.2%	0.87	0.21–3.55	0.847
Subgroup: Children	0	NA	NA	1	NA	NA	NA	NA	NA
Subgroup: Adult	9	3.6%	1.3–6.7%	31	3.9%	2.6–5.4%	0.92	0.38–2.23	0.846
Subgroup: Sample size, ≤ 100	9	3.6%	1.3–6.7%	19	8.3%	3.7–14.2%	0.43	0.15–1.25	0.121
Subgroup: Sample size, >100	0	NA	NA	13	1.5%	0.7–2.4%	NA	NA	NA
Subgroup: Administration of MTX, intramuscularly	1	NA	NA	3	NA	NA	NA	NA	NA
Subgroup: Administration of MTX, orally	0	NA	NA	18	7.3%	3.3–12.4%	NA	NA	NA
Subgroup: Administration of MTX, subcutaneously	2	NA	NA	0	NA	NA	NA	NA	NA
Subgroup: Administration of MTX, combination	6	2.4%	0.1–6.8%	4	NA	NA	NA	NA	NA
Subgroup: Dose of MTX, low	3	NA	NA	19	3.6%	1.3–6.9%	NA	NA	NA
Subgroup: Dose of MTX, high	6	2.2%	0–6.2%	10	4.6%	2.7–7%	0.47	0.04–6.7	0.563
Subgroup: Duration of MTX, short term	5	2.4%	0.1–6.7%	12	1.5%	0.4–3.1%	1.58	0.11–22.97	0.739
Subgroup: Duration of MTX, long term	4	NA	NA	16	4.1%	1.7–7.2%	NA	NA	NA
Subgroup: Usage of steroid, Yes	5	4.1%	1.5–7.6%	6	1.3%	0–4.7%	3.29	0.14–79.43	0.464
Subgroup: Usage of folic acid, Yes	7	3.4%	0.7–7.5%	8	0.7%	0.1–1.5%	5.01	0.96–26.26	0.056
Subgroup: Steroid + low dose + short term	2	NA	NA	2	NA	NA	NA	NA	NA
Subgroup: Steroid + high dose/long term	3	NA	NA	3	NA	NA	NA	NA	NA
Subgroup: Folic acid + low dose + short term	1	NA	NA	3	NA	NA	NA	NA	NA
Subgroup: Folic acid + high dose/long term	6	2.7%	0.1–7.2%	5	1.2%	0.4–2.3%	2.23	0.26–19.2	0.464
Subgroup: Steroid/folic acid + low dose/short term	2	NA	NA	4	NA	NA	NA	NA	NA
Subgroup: Steroid/folic acid + high dose/long term	7	2.9%	0.5–6.6%	8	1.1%	0.3–2.2%	2.59	0.51–13.26	0.253

a *NA indicates that analysis is not performed due to limited number of articles (<5)*.

*IBD, inflammatory bowel disease; CI, confidence interval; RR, relative risk; MTX, methotrexate; NA, not available*.

### Heterogeneity and Sensitivity Analysis

As for TLI, both IBD and non-IBD articles showed considerable heterogeneity (Supplementary Table S1; *I*^2^ = 90 and 97%, respectively). The heterogeneity could not be attenuated in most of the subgroups, with *I*^2^ > 50%. Only in the subgroups of publication date before 2000 and prospective studies, IBD articles showed little heterogeneity (*I*^2^ = 0.4 and 26.9%, respectively). As for MTX-D, IBD articles had little heterogeneity, with *I*^2^ = 27.3% in total and *I*^2^ < 40% in most of the subgroups. While non-IBD articles showed substantial heterogeneity *I*^2^ > 50% in total and in most subgroups. As for LF, the heterogeneity was moderate in IBD articles and substantial in non-IBD articles.

At sensitivity analysis (Supplementary Table S2), the cumulative incidence of TLI was stable when articles with NOS score = 5 were omitted (RR = 1.31; 95% CI, 0.95–1.81). Yet the heterogeneity remained considerable. The risk of MTX-D and LF was higher in patients with IBD (RR = 1.51 and 1.92, respectively). The heterogeneity was little in IBD articles but substantial in non-IBD articles. When articles with NOS score = 6 and 7 were omitted, risk of TLI remained stable (RR = 1.07 and 1.16, respectively). But RR of MTX-D was <1 (RR = 0.87, 95% CI, 0.17–4.57). It should be noted that the number of IBD article with NOS score > 7 was quite limited with only eight articles reporting MTX-D, which might lead to unstable result. The heterogeneity of IBD articles in terms of MTX-D also increased. The number of articles reporting LF was too limited to perform sensitivity analysis.

### Publication Bias

**Figure 2:**
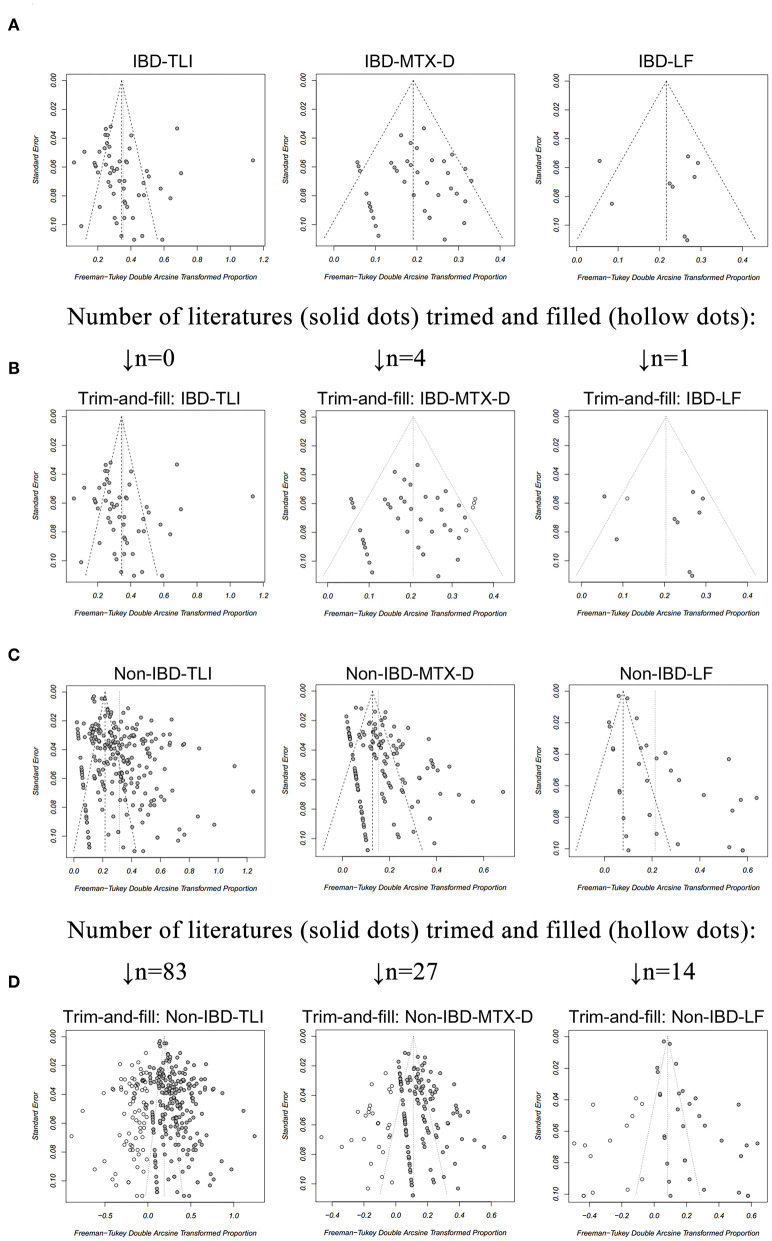
A funnel plot for evaluation of publication bias of **(A)** total liver injury (TLI), methotrexate discontinuation (MTX-D) and liver fibrosis (LF) in patients with inflammatory bowel disease (IBD), receiving MTX treatment; **(B)** TLI, MTX-D, and LF in patients with IBD after the trim-and-fill method; **(C)** TLI, MTX-D, and LF in patients with non-IBD diseases; **(D)** TLI, MTX-D, and LF in patients with non-IBD diseases after the trim-and-fill method.

The funnel plots were found to be almost symmetric for TLI, MTX-D, and LF in IBD (Figure 2A), and publication bias was non-significant (*p* > 0.05 for all; Table 5). Only four and one articles were trimmed and filled in the funnel plots of MTX-D and LF in IBD, respectively (Figure 2B). In contrast, the funnel plots of TLI, MTX-D, and LF in non-IBD articles were obviously asymmetric (Figure 2C). Egger's test also demonstrated significant publication bias (*p* < 0.001 for all; Table 5). Therefore, a total of 83, 27, and 14 articles were trimmed and filled in the funnel plots of TLI, MTX-D, and LF in non-IBD, respectively (Figure 2D). Afterwards, the cumulative incidences of TLI (11.2 vs. 3%; RR = 3.76), MTX-D (3.3 vs 0.7%; RR = 5) and LF (3.1 vs 0.1%; RR = 38.62) in IBD were significantly higher than those in non-IBD (*p* < 0.01 for all; Table 5).

**Table 5:** Publication bias analysis.

	**IBD**				**Non-IBD**						
**Outcome**	**No. of articles**	**Cumulative incidence**	**95% CI**	***P* of Egger's test**	**No. of articles**	**Cumulative incidence**	**95% CI**	***P* of Egger's test**	**RR**	**95% CI of RR**	***P* of RR**
**TLI**											
Original data	54	11.2%	8.1–14.7%	0.604	235	9.2%	8.1–10.3%	<0.001	1.22	0.88–1.68	0.224
After trim-and-fill	54	11.2%	8.1–14.7%	0.604	318	3%	2.3–3.8%	0.256	3.76	2.54–5.55	<0.001
**MTX-D**											
Original data	39	2.6%	1.8- 3.6%	0.775	139	1.8%	1.3–2.3%	<0.001	1.48	0.94–2.33	0.089
After trim-and-fill	43	3.3%	2.2–4.4%	0.852	166	0.7%	0.3–1.1%	0.662	5	2.51–9.98	<0.001
**LF**											
Original data	9	3.6%	1.3–6.7%	NA	32	3.7%	2.5–5.2%	<0.001	0.96	0.39–2.35	0.933
After trim-and-fill	10	3.1%	1.1–5.9%	0.735	46	0.1%	0–0.6%	0.719	38.62	4.17–357.94	0.001

*IBD, inflammatory bowel disease; CI, confidence interval; RR, relative risk; TLI, total liver injury; MTX-D, methotrexate discontinuation; LF, liver fibrosis; NA, not available due to limited number of articles (<10)*.

## Discussion

Liver adverse events are relatively common in the setting of MTX therapy, which can lead to the discontinuation of treatment. Previous reports have indicated that populations with different diseases might have a diverse MTX-related liver toxicity profile. The risk of developing cirrhosis in patients with PsA receiving long-term MTX therapy was found to be high (15); while other research among patients with RA reported lower rates of < 2% (16, 17). The elevation of liver enzymes was reported to be 2.76-fold more likely in patients with PsA compared with RA (18). The risk of liver adverse events in patients with IBD undergoing MTX might be even higher. A previous meta-analysis by Conway et al. (19) included 32 randomized controlled trials, investigating MTX-related liver adverse events in inflammatory diseases (only one study for IBD and 31 studies for RA and Ps/PsA). The cumulative liver adverse events rate was reported to be 11.2%; while the single study for IBD in their analysis showed that 17.5% patients with IBD had elevation of liver enzyme (8). However, there is argument that hepatotoxicity rates do not differ in patients with different diseases treated with MTX (20). The conclusion remains controversial due to limited evidence. In order to further clarify the controversy, we performed this large-scale meta-analysis, including 326 studies with a total of 128,876 patients. In this study, we found a significantly higher risk of liver injury, liver fibrosis, and treatment discontinuation among patients with IBD treated with MTX compared with those with non-IBD diseases.

The characteristics of IBD and non-IBD articles differ in quite a few respects, such as the dose of MTX, concomitant usage of steroid, and sample size. Since MTX is a second-line medication for IBD, there might be diversity in the patient baseline characteristics and therapeutic regimen as compared to non-IBD diseases. For instance, a substantial portion of patients with RA and Ps/PsA receives MTX therapy for more than 4 years, while patients with IBD rarely have long duration of MTX treatment. These factors can lead to distinct different characteristics between IBD and non-IBD articles. More importantly, these differences might have potential effects on the comparison of cumulative incidence of liver injury. Therefore, we performed a thorough exploration of subgroup analysis. With univariate and multivariate adjustment in various subgroups, IBD showed a higher risk of total liver injury and MTX discontinuation, yet the difference was not always significant or close to significant in all subgroups. This might be attributed to the distinct heterogeneity of included studies as well as potential publication bias. Since substantial heterogeneity still existed in most subgroups, especially in non-IBD articles, we assumed that publication bias might be a more critical issue that led to the non-significant statistics. The funnel plots indicated that there is very significant publication bias in non-IBD articles. In line with our consideration, after adjustment by using the trim-and-fill method, the cumulative incidences of liver injury, including TLI, MTX-D, and LF in IBD, were significantly higher than those in non-IBD.

The heterogeneity of non-IBD articles was within our expectations, since the inclusion criteria had no restriction in terms of publication year, the concomitant usage of steroids and folic acid, the dose, as well as the duration of MTX treatment. These factors had influence on the incidence of MTX toxicity and could lead to heterogeneity. In several subgroups with the restriction of variables, as well as decreased number of studies, the heterogeneity could be reduced. Besides, we found significant publication bias in non-IBD articles. The bias was mainly due to over-reported high incidence of liver injury and less reports with low incidence. The bias existed in non-IBD, but not IBD articles might be due to longer term of MTX as well as the less usage of steroids in non-IBD. Besides, during the full-text screening process, we observed that many non-IBD articles, especially those with small sample size and short term of MTX, did not report the liver adverse events, instead of reporting “zero-liver adverse events” (21). Therefore, quite a few non-IBD articles with low or zero incidence of liver injury might be missed, which could further increase the publication bias. Thus, the results after trim-and-fill might be more reasonable and credible.

A previous report indicated that a lower dose of MTX in the treatment of inflammatory diseases might be associated with a lower risk of liver injury (22, 23) as compared with high dose of MTX (13, 24). In the present meta-analysis, most of the included non-IBD articles (63.4%) used low dose of MTX, while more IBD articles (66.7%) used high-dose MTX. This could lead to a potential bias effect on the overall incidence of liver injury in IBD and non-IBD diseases treated by MTX. Therefore, we have performed a comprehensive subgroup analysis in terms of the dose and the duration of MTX. The results showed that, in either low- or high-dose MTX subgroups, there was a higher incidence of TLI in patients with IBD as compared with non-IBD (RR > 1). The similar results were observed in the subgroups of short-term or long-term MTX (Table 1). Thus, the conclusion remains unchanged after adjusting the potential confounding factors. As for the risk of MTX-D, a higher risk was also observed in each of the subgroups, including low- or high-dose MTX and short-term or long-term MTX (Table 2). The risk of LF is difficult to be evaluated in subgroup analysis due to the limited number of articles, which had available data on liver fibrosis. Taken together, the results of subgroup analysis further supported the main conclusion. On the other hand, there is significant difference in the distribution of prospective and retrospective studies between IBD articles and non-IBD articles (*p* < 0.001; Table 1). The superiority of prospective study is that the detailed data of exposure (MTX treatment) are collected before the outcome of the liver injury is known and, therefore, cannot be biased by the outcome. Therefore, the result in the subgroup of prospective studies might be more accurate. Notably, the difference of MTX-related TLI risk between IBD and non-IBD articles reached the highest statistical significance in the subgroup of prospective studies (14.3 vs. 8.9%, RR = 1.62, *P* = 0.007). Thus, the result in prospective studies further strengthens the main conclusion of this meta-analysis.

The mechanism of different risks of liver injury in IBD and non-IBD diseases deserves further investigation *via* clinical and basic research. It is known that the main mechanism of MTX effect is related with the direct inhibition of DHFR and other key enzymes associated with folate metabolism (25). DHFR inhibition can mediate the disruption of DNA replication and subsequent cell death. Polymorphism rs1650697 (C35T) resides in a major promoter of *DHFR* gene (26), which is responsible for 99% transcription of the gene. It has been reported that C35T polymorphism is associated with the incidence of hepatotoxicity in patients with RA receiving MTX treatment. The presence of T allele might be protective against MTX hepatotoxicity (27). Besides, other gene polymorphisms, such as C677T and A1298C polymorphisms in the *MTHFR* gene, are also related to the toxicity of MTX in patients with RA (28, 29). It was reported that the variant allele in *MTHFR* A1298C was significantly higher in patients with IBD (30). Thus, further exploring the polymorphism profiles of relevant genes in patients with IBD and non-IBD diseases might provide better understanding of the different risks of liver injury. On the other hand, the liver is closely associated with the gut *via* the gut-liver axis (31). The gut dysbiosis might lead to a disturbed hepatic metabolism, which is involved in drug-induced hepatotoxicity (32). Furthermore, in our preliminary experiment (33), we also found that, for rats receiving hepatotoxic reagent (carbon tetrachloride), the severity of liver injury could be significantly aggravated by the experimental colitis model (induced by dextran sulfate sodium). Therefore, the gut-liver axis might also be a potential trigger for the high incidence of MTX-related liver injury in patients with IBD.

One limitation of the present report is that the large number of included articles could lead to increased heterogeneity, and the heterogeneity cannot be effectively eliminated by subgroup analysis. Currently, this issue is difficult to solve since few clinical trials are designed to compare the safety of MTX in patients with IBD and non-IBD diseases. Thus, we have to include single arm studies. Nevertheless, the heterogeneity might not be a major confounding factor to the results, since the RR values were > 1 in most subgroups, and the difference was more significant after adjustment for publication bias.

## Conclusion

The results of the present meta-analysis demonstrate a higher risk of liver injury in patients with IBD, receiving MTX treatment, compared with patients with non-IBD diseases. The heterogeneity of the included studies is a limitation, but the available data provide robust evidence for the conclusion. Future clinical and basic investigations are necessary to clarify the mechanism of MTX-induced liver injury in IBD.

## Data Availability Statement

The original contributions presented in the study are included in the article/Supplementary Materials, further inquiries can be directed to the corresponding author.

## Author Contributions

YW and YulL proposed the study concept and design. YW and YimL collected the literatures and extracted the data. Dissonance was resolved with YunL and ZK by discussion. YW and YimL did the data analysis and drafted the manuscript. YW and YiZ contributed to the interpretation of data. YW, YunL, YuZ, and YulL discussed the significance of this research. YulL critically revised the manuscript. All authors contributed to the article and approved the submitted version.

## Funding

This work was supported by the National Natural Science Foundation of China (Grant Numbers: 82070539 and 81873549). The sponsors had no involvement in the collection, analysis and interpretation of data; in the writing of the report; and in the decision to submit the article for publication.

## Conflict of Interest

The authors declare that the research was conducted in the absence of any commercial or financial relationships that could be construed as a potential conflict of interest.

## Publisher's Note

All claims expressed in this article are solely those of the authors and do not necessarily represent those of their affiliated organizations, or those of the publisher, the editors and the reviewers. Any product that may be evaluated in this article, or claim that may be made by its manufacturer, is not guaranteed or endorsed by the publisher.

## Abbreviations

MTX: methotrexate
DHFR: dihydrofolate reductase
IBD: inflammatory bowel disease
RA: rheumatoid arthritis
Ps: psoriasis
PsA: psoriatic arthritis
ALT: alanine aminotransferase
ULN: upper limit of normal
TLI: total liver injury
MTX-D: MTX discontinuation
LF: liver fibrosis
NOS: Newcastle-Ottawa Scale
RR: relative risk.
