# Supplementary figures and images for: Patients With IBD Receiving Methotrexate Are at Higher Risk of Liver Injury Compared With Patients With Non-IBD Diseases: A Meta-Analysis and Systematic Review
Source: Front Med (Lausanne). 2021 Nov 22;8:774824. doi: 10.3389/fmed.2021.774824 (PMC8645797; doi:10.3389/fmed.2021.774824)

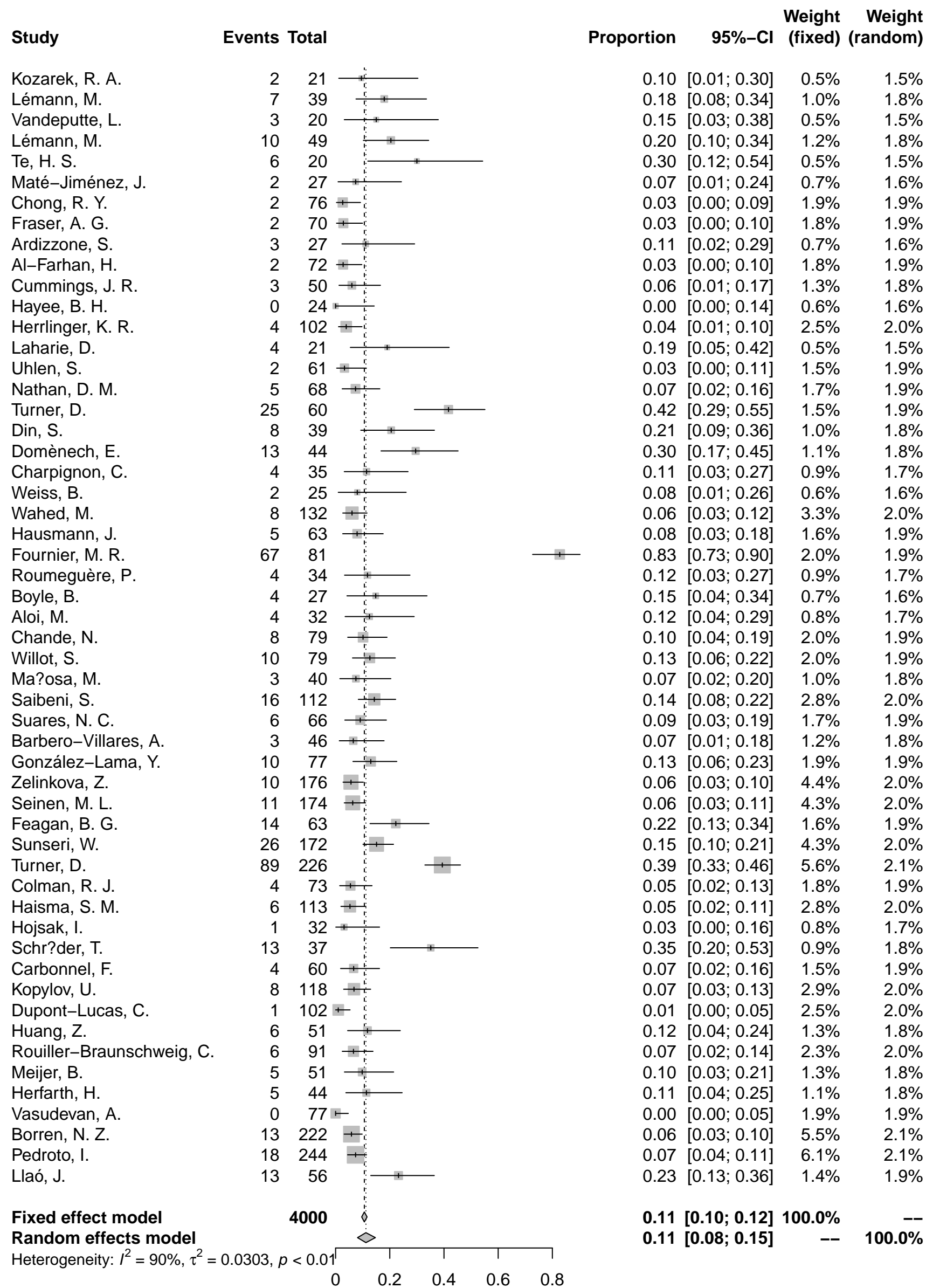

Supplement: Supplementary file 1 [file Image_1.pdf]

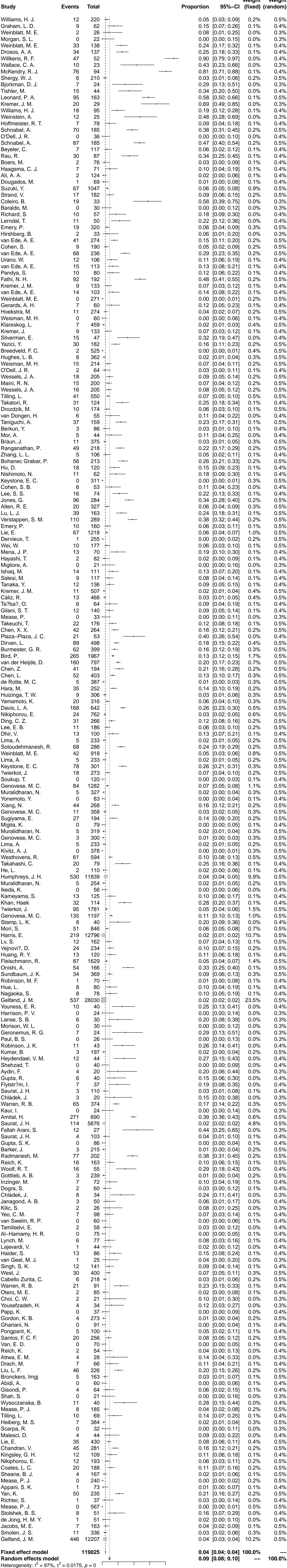

Supplement: Supplementary file 2 [file Image_2.pdf]

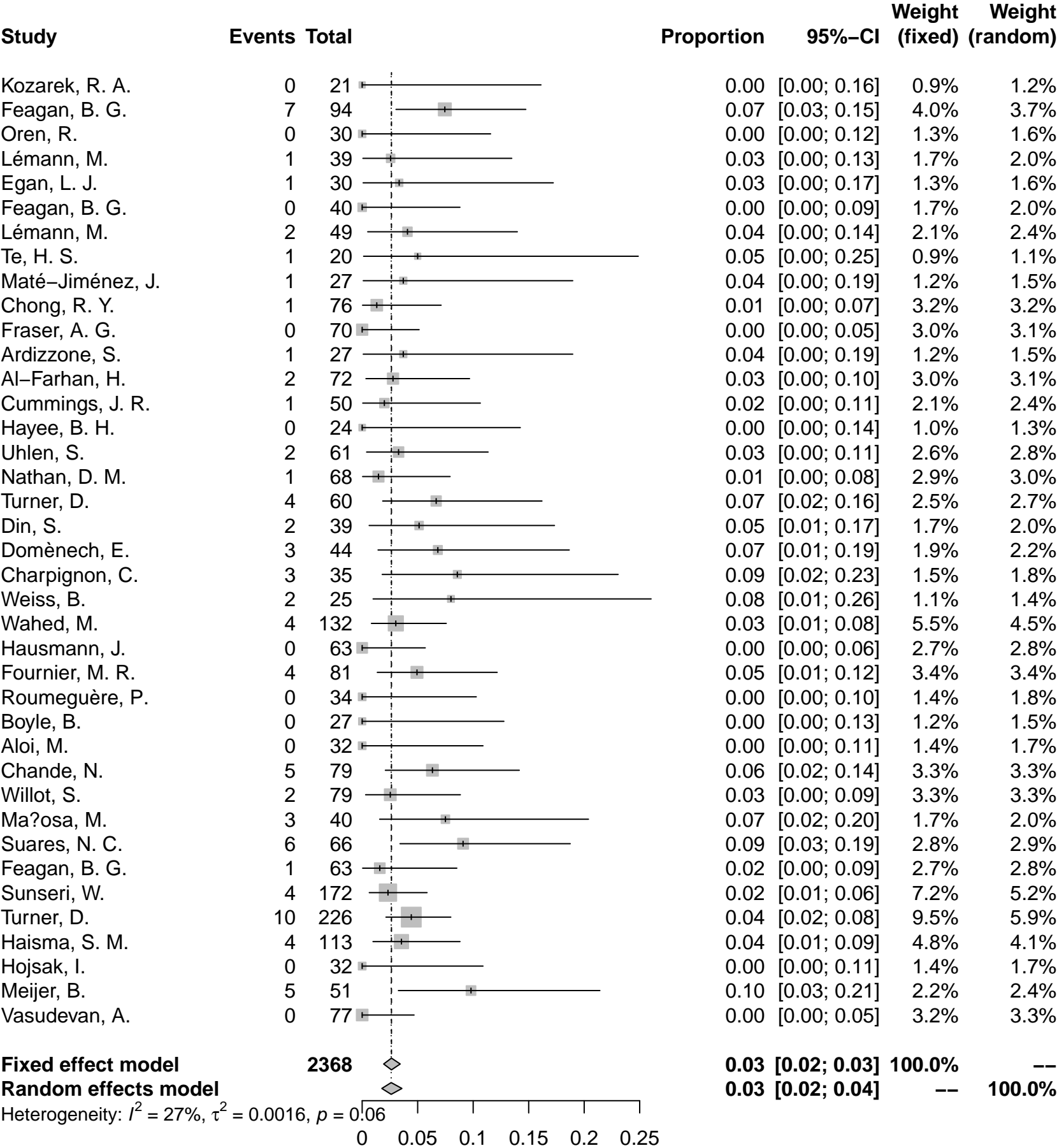

Supplement: Supplementary file 3 [file Image_3.pdf]

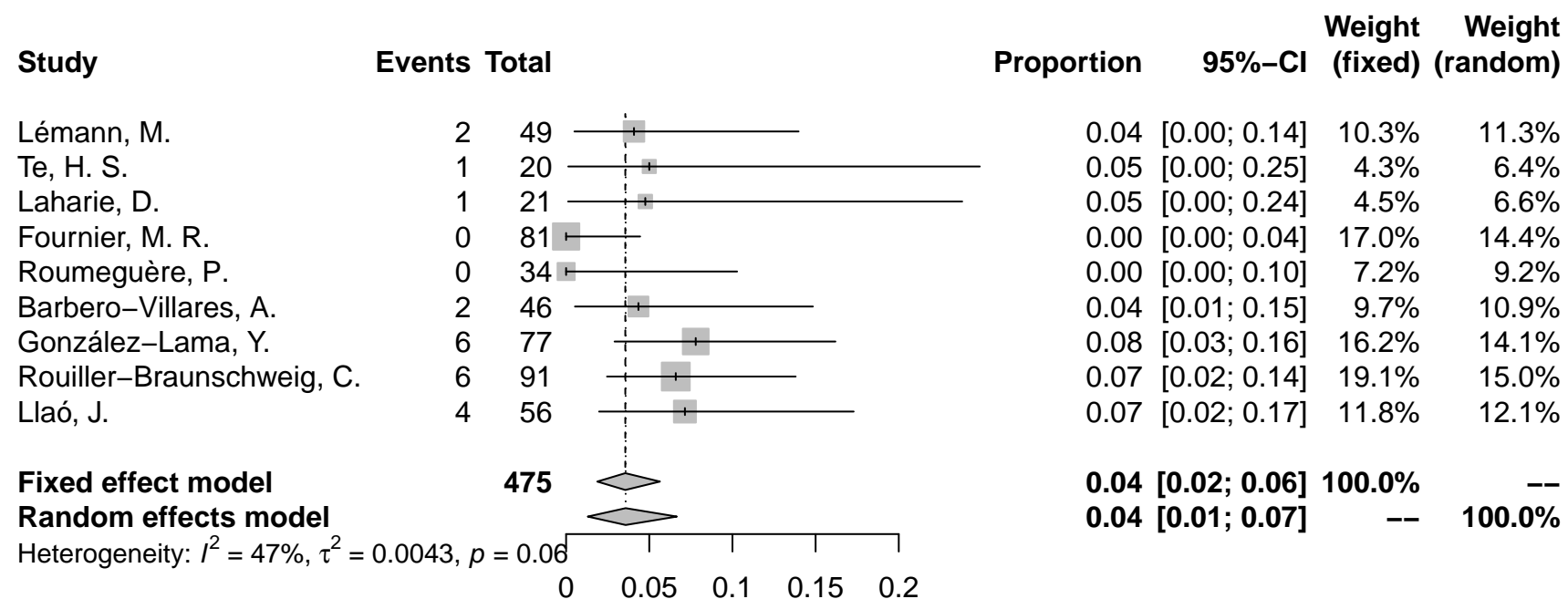

Supplement: Supplementary file 5 [file Image_5.pdf]

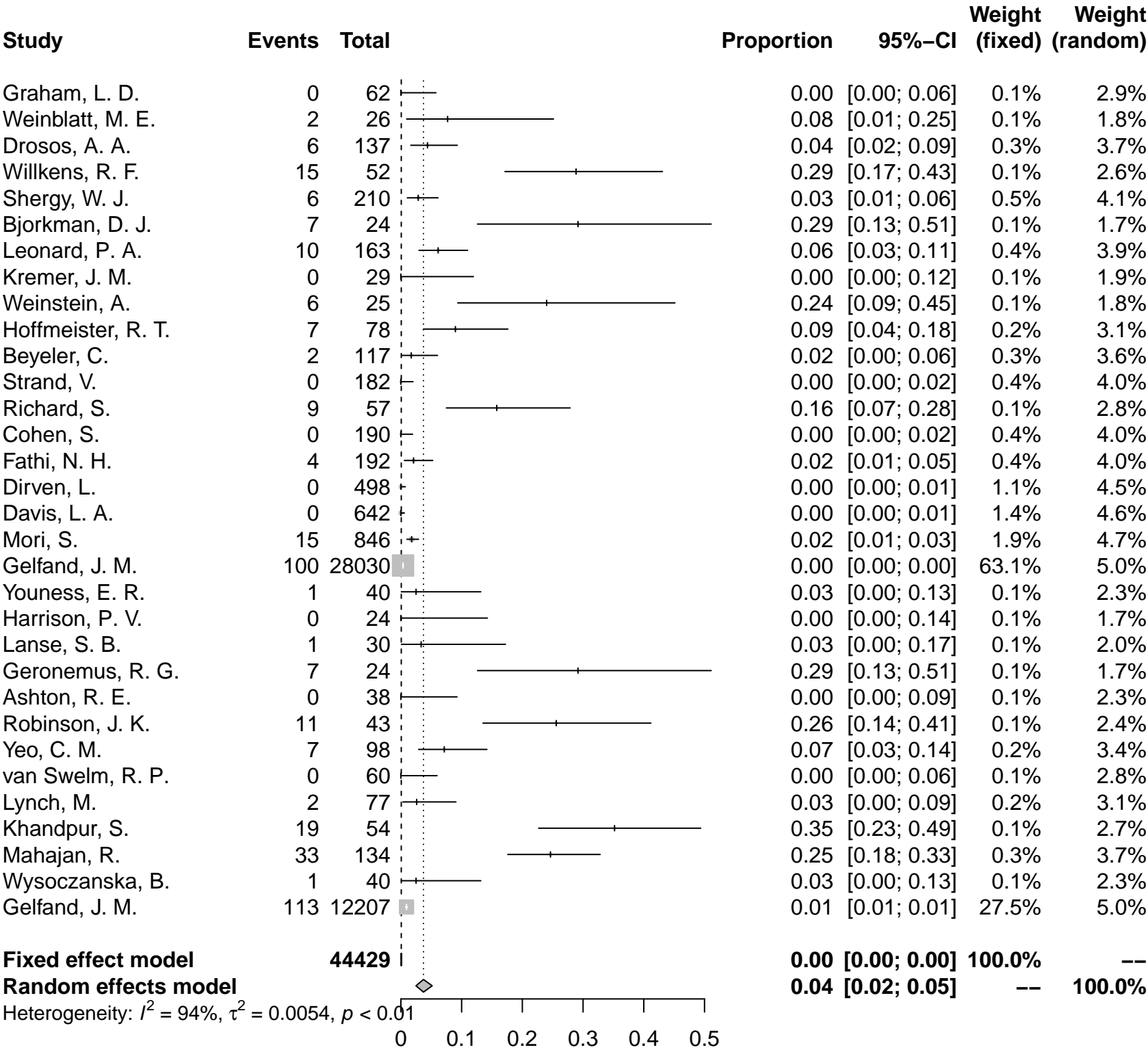

Supplement: Supplementary file 6 [file Image_6.pdf]
