# Supplementary material for: Patients With IBD Receiving Methotrexate Are at Higher Risk of Liver Injury Compared With Patients With Non-IBD Diseases: A Meta-Analysis and Systematic Review
Source: Front Med (Lausanne). 2021 Nov 22;8:774824. doi: 10.3389/fmed.2021.774824 (PMC8645797; doi:10.3389/fmed.2021.774824)

Study	Events	Total	Proportion	95%−CI	Weight (fixed)	Weight (random)
Williams, H. J.	12	220	0.05	[0.03; 0.09]	0.8%	0.8%
Willkens, R. F.	2	136	0.01	[0.00; 0.05]	0.5%	0.8%
Weinblatt, M. E.	1	26	0.04	[0.00; 0.20]	0.1%	0.4%
Weinblatt, M. E.	6	138	0.04	[0.02; 0.09]	0.5%	0.8%
Drosos, A. A.	6	137	0.04	[0.02; 0.09]	0.5%	0.8%
Wallace, C. A.	3	23	0.13	[0.03; 0.34]	0.1%	0.4%
McKendry, R. J.	13	94	0.14	[0.08; 0.22]	0.3%	0.7%
Shergy, W. J.	5	210	0.02	[0.01; 0.05]	0.7%	0.8%
Tishler, M.	0	44	0.00	[0.00; 0.08]	0.2%	0.6%
Leonard, P. A.	8	163	0.05	[0.02; 0.09]	0.6%	0.8%
Kremer, J. M.	0	29	0.00	[0.00; 0.12]	0.1%	0.5%
Williams, H. J.	18	95	0.19	[0.12; 0.28]	0.3%	0.7%
Weinstein, A.	0	25	0.00	[0.00; 0.14]	0.1%	0.4%
Hoffmeister, R. T.	2	78	0.03	[0.00; 0.09]	0.3%	0.7%
O'Dell, J. R.	0	36	0.00	[0.00; 0.10]	0.1%	0.5%
Schnabel, A.	4	185	0.02	[0.01; 0.05]	0.7%	0.8%
Boers, M.	0	76	0.00	[0.00; 0.05]	0.3%	0.7%
Haagsma, C. J.	7	71	0.10	[0.04; 0.19]	0.3%	0.7%
Strand, V.	8	182	0.04	[0.02; 0.08]	0.6%	0.8%
Coleiro, B.	4	33	0.12	[0.03; 0.28]	0.1%	0.5%
Baraldo, M.	0	30	0.00	[0.00; 0.12]	0.1%	0.5%
Lerndal, T.	11	50	0.22	[0.12; 0.36]	0.2%	0.6%
Emery, P.	5	320	0.02	[0.01; 0.04]	1.1%	0.9%
Hirshberg, B.	0	33	0.00	[0.00; 0.11]	0.1%	0.5%
van Ede, A. E.	11	274	0.04	[0.02; 0.07]	1.0%	0.9%
van Ede, A. E.	30	236	0.13	[0.09; 0.18]	0.8%	0.9%
van Ede, A. E.	15	113	0.13	[0.08; 0.21]	0.4%	0.8%
Pandya, S.	0	80	0.00	[0.00; 0.05]	0.3%	0.7%
Fathi, N. H.	0	192	0.00	[0.00; 0.02]	0.7%	0.8%
Kremer, J. M.	0	133	0.00	[0.00; 0.03]	0.5%	0.8%
van Ede, A. E.	14	103	0.14	[0.08; 0.22]	0.4%	0.7%
Weinblatt, M. E.	0	271	0.00	[0.00; 0.01]	1.0%	0.9%
Weisman, M. H.	0	60	0.00	[0.00; 0.06]	0.2%	0.6%
Kremer, J.	2	133	0.02	[0.00; 0.05]	0.5%	0.8%
Silverman, E.	1	47	0.02	[0.00; 0.11]	0.2%	0.6%
Yazici, Y.	0	182	0.00	[0.00; 0.02]	0.6%	0.8%
O'Dell, J. R.	2	64	0.03	[0.00; 0.11]	0.2%	0.6%
Wessels, J. A.	3	205	0.01	[0.00; 0.04]	0.7%	0.8%
Maini, R. N.	5	200	0.03	[0.01; 0.06]	0.7%	0.8%
Tilling, L.	7	550	0.01	[0.01; 0.03]	2.0%	0.9%
Takatori, R.	4	124	0.03	[0.01; 0.08]	0.4%	0.8%
Drozdzik, M.	10	174	0.06	[0.03; 0.10]	0.6%	0.8%
van Dongen, H.	0	55	0.00	[0.00; 0.06]	0.2%	0.6%
Mor, A.	0	44	0.00	[0.00; 0.08]	0.2%	0.6%
Nishimoto, N.	0	62	0.00	[0.00; 0.06]	0.2%	0.6%
Keystone, E. C.	0	311	0.00	[0.00; 0.01]	1.1%	0.9%
Cohen, S. B.	3	53	0.06	[0.01; 0.16]	0.2%	0.6%
Lee, S. S.	2	74	0.03	[0.00; 0.09]	0.3%	0.7%
Jones, G.	2	284	0.01	[0.00; 0.03]	1.0%	0.9%
Alten, R. E.	2	327	0.01	[0.00; 0.02]	1.2%	0.9%
Verstappen, S. M.	7	289	0.02	[0.01; 0.05]	1.0%	0.9%
Lie, E.	40	1218	0.03	[0.02; 0.04]	4.3%	1.0%
Dervieux, T.	0	255	0.00	[0.00; 0.01]	0.9%	0.9%
Migliore, A.	0	21	0.00	[0.00; 0.16]	0.1%	0.4%
Salesi, M.	0	117	0.00	[0.00; 0.03]	0.4%	0.8%
Tanaka, Y.	4	136	0.03	[0.01; 0.07]	0.5%	0.8%
Kremer, J. M.	6	507	0.01	[0.00; 0.03]	1.8%	0.9%
Cáliz, R.	13	468	0.03	[0.01; 0.05]	1.7%	0.9%
Mease, P.	0	33	0.00	[0.00; 0.11]	0.1%	0.5%
Takeuchi, T.	2	176	0.01	[0.00; 0.04]	0.6%	0.8%
Chen, X. X.	0	264	0.00	[0.00; 0.01]	0.9%	0.9%
Plaza−Plaza, J. C.	21	53	0.40	[0.26; 0.54]	0.2%	0.6%
Dirven, L.	9	498	0.02	[0.01; 0.03]	1.8%	0.9%
Burmester, G. R.	0	399	0.00	[0.00; 0.01]	1.4%	0.9%
Bird, P.	6	1967	0.00	[0.00; 0.01]	7.0%	1.0%
Chen, Z.	0	194	0.00	[0.00; 0.02]	0.7%	0.8%
Hara, M.	1	252	0.00	[0.00; 0.02]	0.9%	0.9%
Davis, L. A.	17	642	0.03	[0.02; 0.04]	2.3%	0.9%
Nikiphorou, E.	24	762	0.03	[0.02; 0.05]	2.7%	0.9%
Sotoudehmanesh, R.	1	286	0.00	[0.00; 0.02]	1.0%	0.9%
Keystone, E. C.	0	301	0.00	[0.00; 0.01]	1.1%	0.9%
?wierkot, J.	5	273	0.02	[0.01; 0.04]	1.0%	0.9%
Genovese, M. C.	24	1282	0.02	[0.01; 0.03]	4.5%	1.0%
Muralidharan, N.	5	327	0.02	[0.00; 0.04]	1.2%	0.9%
Yonemoto, Y.	0	83	0.00	[0.00; 0.04]	0.3%	0.7%
Migita, K.	0	79	0.00	[0.00; 0.05]	0.3%	0.7%
Muralidharan, N.	5	319	0.02	[0.01; 0.04]	1.1%	0.9%
Genovese, M. C.	0	300	0.00	[0.00; 0.01]	1.1%	0.9%
Kivitz, A. J.	0	378	0.00	[0.00; 0.01]	1.3%	0.9%
Muralidharan, N.	5	254	0.02	[0.01; 0.05]	0.9%	0.9%
Ikeda, K.	0	56	0.00	[0.00; 0.06]	0.2%	0.6%
?wierkot, J.	11	1781	0.01	[0.00; 0.01]	6.3%	1.0%
Mori, S.	0	846	0.00	[0.00; 0.00]	3.0%	0.9%
Vejnovi?, D.	0	234	0.00	[0.00; 0.02]	0.8%	0.9%
Huang, R. Y.	2	120	0.02	[0.00; 0.06]	0.4%	0.8%
Robinson, M. F.	0	70	0.00	[0.00; 0.05]	0.2%	0.7%
Youness, E. R.	3	40	0.07	[0.02; 0.20]	0.1%	0.5%
Harrison, P. V.	0	24	0.00	[0.00; 0.14]	0.1%	0.4%
Lanse, S. B.	1	30	0.03	[0.00; 0.17]	0.1%	0.5%
Morison, W. L.	0	30	0.00	[0.00; 0.12]	0.1%	0.5%
Paul, B. S.	0	26	0.00	[0.00; 0.13]	0.1%	0.4%
Heydendael, V. M.	12	44	0.27	[0.15; 0.43]	0.2%	0.6%
Shehzad, T.	0	40	0.00	[0.00; 0.09]	0.1%	0.5%
Flytstr?m, I.	0	37	0.00	[0.00; 0.09]	0.1%	0.5%
Saurat, J. H.	3	110	0.03	[0.01; 0.08]	0.4%	0.7%
Kaur, I.	0	24	0.00	[0.00; 0.14]	0.1%	0.4%
Fallah Arani, S.	2	27	0.07	[0.01; 0.24]	0.1%	0.4%
Saurat, J. H.	3	103	0.03	[0.01; 0.08]	0.4%	0.7%
Gupta, S. K.	0	86	0.00	[0.00; 0.04]	0.3%	0.7%
Barker, J.	3	215	0.01	[0.00; 0.04]	0.8%	0.8%
Reich, K.	2	163	0.01	[0.00; 0.04]	0.6%	0.8%
Woolf, R. T.	3	55	0.05	[0.01; 0.15]	0.2%	0.6%
Inzinger, M.	7	72	0.10	[0.04; 0.19]	0.3%	0.7%
Dogra, S.	2	60	0.03	[0.00; 0.12]	0.2%	0.6%
Tamilselvi, E.	2	58	0.03	[0.00; 0.12]	0.2%	0.6%
Al−Hamamy, H. R.	0	75	0.00	[0.00; 0.05]	0.3%	0.7%
Lynch, M.	2	77	0.03	[0.00; 0.09]	0.3%	0.7%
Lajevardi, V.	1	44	0.02	[0.00; 0.12]	0.2%	0.6%
Haider, S.	13	86	0.15	[0.08; 0.24]	0.3%	0.7%
van Geel, M. J.	1	25	0.04	[0.00; 0.20]	0.1%	0.4%
Singh, S. K.	7	141	0.05	[0.02; 0.10]	0.5%	0.8%
West, J.	30	400	0.07	[0.05; 0.11]	1.4%	0.9%
Cabello Zurita, C.	6	218	0.03	[0.01; 0.06]	0.8%	0.8%
Warren, R. B.	11	91	0.12	[0.06; 0.21]	0.3%	0.7%
Otero, M. E.	2	85	0.02	[0.00; 0.08]	0.3%	0.7%
Papp, K.	0	37	0.00	[0.00; 0.09]	0.1%	0.5%
Ghariani, N.	0	91	0.00	[0.00; 0.04]	0.3%	0.7%
Pongparit, K.	5	100	0.05	[0.02; 0.11]	0.4%	0.7%
Santos, F. C. F.	0	256	0.00	[0.00; 0.01]	0.9%	0.9%
Van, E. D.	0	70	0.00	[0.00; 0.05]	0.2%	0.7%
Abidi, A.	0	60	0.00	[0.00; 0.06]	0.2%	0.6%
Gisondi, P.	4	64	0.06	[0.02; 0.15]	0.2%	0.6%
Shah, S.	0	21	0.00	[0.00; 0.16]	0.1%	0.4%
Wysoczanska, B.	0	40	0.00	[0.00; 0.09]	0.1%	0.5%
Mease, P. J.	1	185	0.01	[0.00; 0.03]	0.7%	0.8%
Tilling, L.	4	69	0.06	[0.02; 0.14]	0.2%	0.7%
Heiberg, M. S.	7	384	0.02	[0.01; 0.04]	1.4%	0.9%
Scarpa, R.	0	32	0.00	[0.00; 0.11]	0.1%	0.5%
Malesci, D.	4	44	0.09	[0.03; 0.22]	0.2%	0.6%
Lie, E.	20	430	0.05	[0.03; 0.07]	1.5%	0.9%
Nikiphorou, E.	12	193	0.06	[0.03; 0.11]	0.7%	0.8%
Coates, L. C.	4	188	0.02	[0.01; 0.05]	0.7%	0.8%
Sheane, B. J.	4	167	0.02	[0.01; 0.06]	0.6%	0.8%
Mease, P. J.	0	240	0.00	[0.00; 0.02]	0.9%	0.9%
Appani, S. K.	0	73	0.00	[0.00; 0.05]	0.3%	0.7%
Yan, K.	0	235	0.00	[0.00; 0.02]	0.8%	0.9%
Mease, P. J.	0	567	0.00	[0.00; 0.01]	2.0%	0.9%
Stolshek, B. S.	8	51	0.16	[0.07; 0.29]	0.2%	0.6%
de Jong, H. M. Y.	1	51	0.02	[0.00; 0.10]	0.2%	0.6%
**Fixed effect model**		**28159**	**0.01**	**[0.01; 0.01]**	**100.0%**	**−−**
**Random effects model**			**0.02**	**[0.01; 0.02]**	**−−**	**100.0%**

Heterogeneity: $I^2 = 85\%$, $\tau^2 = 0.0072$, $p < 0.01$

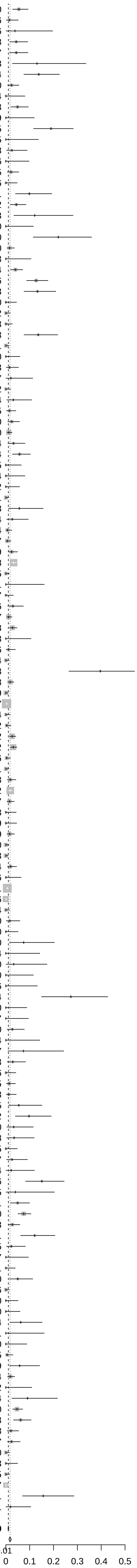

Supplement: Supplementary file 4 [file Image_4.pdf]
