# Supplementary material for: Patients With IBD Receiving Methotrexate Are at Higher Risk of Liver Injury Compared With Patients With Non-IBD Diseases: A Meta-Analysis and Systematic Review
Source: Front Med (Lausanne). 2021 Nov 22;8:774824. doi: 10.3389/fmed.2021.774824 (PMC8645797; doi:10.3389/fmed.2021.774824)
Supplement: Supplementary file 7 [file Table_6.DOCX]

**Table S1. Heterogeneity of included articles**

|  | IBD | | | | | |  | Non-IBD diseases | | | | | |
| --- | --- | --- | --- | --- | --- | --- | --- | --- | --- | --- | --- | --- | --- |
| Groups | TLI:  No. of ariticles | TLI:  *I^2^* | MTX-D:  No. of ariticles | MTX-D:  *I^2^* | LF:  No. of ariticles | LF:  *I^2^* |  | TLI:  No. of ariticles | TLI:  *I^2^* | MTX-D:  No. of ariticles | MTX-D:  *I^2^* | LF:  No. of ariticles | LF:  *I^2^* |
| Total | 54 | 90% | 39 | 27.3% | 9 | 47.1% |  | 235 | 97% | 139 | 85.2% | 32 | 94.2% |
| Subgroup:  Year of publication, before 2000 | 6 | 0.4% | 9 | 0% | 2 | NA |  | 42 | 96.3% | 30 | 72.5% | 20 | 85.7% |
| Subgroup:  Year of publication, after 2000 | 48 | 90.9% | 30 | 34.2% | 7 | 59.7% |  | 193 | 96.8% | 109 | 85.9% | 12 | 96% |
| Subgroup:  Region of study, North America | 14 | 95.5% | 13 | 4.5% | 2 | NA |  | 60 | 98% | 41 | 82.4% | 17 | 93.4% |
| Subgroup:  Region of study, Europe | 35 | 64% | 21 | 32.9% | 7 | 0% |  | 93 | 95.8% | 57 | 88.1% | 10 | 81.1% |
| Subgroup:  Study design, prospective | 10 | 26.9% | 10 | 7.9% | 4 | NA |  | 149 | 95.4% | 95 | 84.7% | 15 | 94% |
| Subgroup:  Study design, retrospective | 44 | 91.5% | 29 | 34.1% | 5 | 65.3% |  | 86 | 97.7% | 44 | 85.7% | 17 | 94.2% |
| Subgroup: Children | 11 | 93.4% | 10 | 0% | 0 | NA |  | 6 | 88.5% | 5 | 39.5% | 1 | NA |
| Subgroup: Adult | 43 | 88.1% | 29 | 36.3% | 9 | 47.1% |  | 229 | 97.1% | 134 | 85.6% | 31 | 94.4% |
| Subgroup: Sample size, <=100 | 42 | 88.4% | 35 | 33.4% | 9 | 47.1% |  | 99 | 91.6% | 66 | 78.4% | 19 | 85.8% |
| Subgroup: Sample size, >100 | 12 | 93.3% | 4 | NA | 0 | NA |  | 136 | 97.9% | 73 | 87.4% | 13 | 94.8% |
| Subgroup: Dose of MTX, low | 17 | 89.1% | 13 | 15.6% | 3 | NA |  | 153 | 94.2% | 95 | 81.9% | 19 | 87.9% |
| Subgroup: Dose of MTX, high | 36 | 90.6% | 26 | 33.2% | 6 | 51.4% |  | 66 | 97.8% | 38 | 85.6% | 10 | 96.4% |
| Subgroup:  Duration of MTX, short term | 42 | 90.2% | 34 | 16.9% | 5 | 64.1% |  | 190 | 96.2% | 115 | 85.7% | 12 | 90.3% |
| Subgroup:  Duration of MTX, long term | 10 | 90.1% | 5 | 66.1% | 4 | NA |  | 38 | 98.2% | 22 | 83.1% | 16 | 90% |
| Subgroup: Usage of steroid, Yes | 37 | 83.2% | 30 | 17.7% | 5 | 5.6% |  | 56 | 96.1% | 34 | 71.5% | 6 | 86.5% |
| Subgroup: Usage of folic acid, Yes | 33 | 90.6% | 21 | 4.7% | 7 | 60.2% |  | 113 | 96.5% | 64 | 85.9% | 8 | 94.9% |
| Subgroup:  Steroid + low dose + short term | 11 | 91.2% | 9 | 0% | 2 | NA |  | 31 | 89.2% | 21 | 69.6% | 2 | NA |
| Subgroup:  Steroid + high dose/long term | 26 | 71.8% | 21 | 27.2% | 3 | NA |  | 22 | 97.7% | 11 | 67.1% | 3 | NA |
| Subgroup:  Folic acid + low dose + short term | 8 | 68.7% | 6 | 0% | 1 | NA |  | 56 | 86.9% | 32 | 81.7% | 3 | NA |
| Subgroup:  Folic acid + high dose/long term | 25 | 92.2% | 15 | 14.5% | 6 | 60.4% |  | 53 | 97.8% | 30 | 82.1% | 5 | 97% |
| Subgroup:  Steroid/folic acid + low dose/short term | 12 | 91.2% | 10 | 0% | 2 | NA |  | 71 | 88.5% | 44 | 80.9% | 4 | NA |
| Subgroup:  Steroid/folic acid + high dose/long term | 34 | 90.1% | 24 | 24.9% | 7 | 53.1% |  | 66 | 98.1% | 38 | 80.5% | 8 | 95.2% |

Abbreviations:IBD, inflammatory bowel disease; TLI, total liver injury; MTX-D, methotrexate discontinuation; LF, liver fibrosis.
