# Supplementary material for: Patients With IBD Receiving Methotrexate Are at Higher Risk of Liver Injury Compared With Patients With Non-IBD Diseases: A Meta-Analysis and Systematic Review
Source: Front Med (Lausanne). 2021 Nov 22;8:774824. doi: 10.3389/fmed.2021.774824 (PMC8645797; doi:10.3389/fmed.2021.774824)
Supplement: Supplementary file 8 [file Table_7.DOCX]

**Table S2.** **Sensitivity analysis**

|  | IBD | | | |  | Non-IBD | | | |  |  |  |
| --- | --- | --- | --- | --- | --- | --- | --- | --- | --- | --- | --- | --- |
| Groups | No. of articles | Cumulative incidence | 95% CI | *I*^2^ |  | No. of articles | Cumulative incidence | 95% CI | *I*^2^ | RR | 95% CI of RR | P of RR |
| Excluded articles:NOS 5 |  |  |  |  |  |  |  |  |  |  |  |  |
| TLI | 52 | 11.4% | 8.2 - 15% | 90.2% |  | 227 | 8.7% | 7.7 - 9.8% | 96.9% | 1.31 | 0.95 - 1.81 | 0.105 |
| MTX-D | 37 | 2.7% | 1.8 - 3.7% | 29% |  | 136 | 1.8% | 1.3 - 2.3% | 85.4% | 1.51 | 0.95 - 2.39 | 0.078 |
| LF | 8 | 4.2% | 1.7 - 7.4% | 44.3% |  | 26 | 2.2% | 1.3 - 3.3% | 92.8% | 1.92 | 0.8 - 4.62 | 0.145 |
| Excluded articles:NOS 5+6 |  |  |  |  |  |  |  |  |  |  |  |  |
| TLI | 12 | 9.4% | 5.3 - 14.5% | 76.2% |  | 65 | 8.8% | 6.4 - 11.6% | 96% | 1.07 | 0.6 - 1.92 | 0.817 |
| MTX-D | 8 | 2.3% | 0.2 - 5.6% | 58.6% |  | 40 | 2.6% | 1.5 - 4.1% | 89.1% | 0.87 | 0.17 - 4.57 | 0.87 |
| LF | 2 | NA | NA | NA |  | 2 | NA | NA | NA | NA | NA | NA |
| Excluded articles:NOS 5+6+7 |  |  |  |  |  |  |  |  |  |  |  |  |
| TLI | 11 | 10.2% | 5.6 - 15.8% | 76.3% |  | 63 | 8.8% | 6.2 - 11.8% | 96.1% | 1.16 | 0.63 - 2.14 | 0.635 |
| MTX-D | 8 | 2.3% | 0.2 - 5.6% | 58.6% |  | 40 | 2.6% | 1.5 - 4.1% | 89.1% | 0.87 | 0.17 - 4.57 | 0.87 |
| LF | 2 | NA | NA | NA |  | 2 | NA | NA | NA | NA | NA | NA |

Abbreviations:IBD, inflammatory bowel disease; CI, confidence interval; RR, relative risk; NOS, Newcastle-Ottawa Scale; TLI, total liver injury; MTX-D, methotrexate discontinuation; LF, liver fibrosis; NA, not available.
