# Supplementary material for: Patients With IBD Receiving Methotrexate Are at Higher Risk of Liver Injury Compared With Patients With Non-IBD Diseases: A Meta-Analysis and Systematic Review
Source: Front Med (Lausanne). 2021 Nov 22;8:774824. doi: 10.3389/fmed.2021.774824 (PMC8645797; doi:10.3389/fmed.2021.774824)
Supplement: Supplementary file 9 [file Table_8.DOCX]

**Supplementary References**

**Included articles of inflammatory bowel disease:**

1. Al-Farhan H, Al-Darmaki AK, Pang JXQ, Fedorak RN, Devlin SM, Dieleman LA, Kaplan GG, et al. Experience with the use of low-dose methotrexate for inflammatory bowel disease. J Can Assoc Gastroenterol 2004;16:921-926.

2. Aloi M, Di Nardo G, Conte F, Mazzeo L, Cavallari N, Nuti F, Cucchiara S, et al. Methotrexate in paediatric ulcerative colitis: a retrospective survey at a single tertiary referral centre. Aliment Pharmacol Ther 2010;32:1017-1022.

3. Ardizzone S, Bollani S, Manzionna G, Imbesi V, Colombo E, Bianchi Porro G. Comparison between methotrexate and azathioprine in the treatment of chronic active Crohn's disease: a randomised, investigator-blind study. Dig Liver Dis 2003;35:619-627.

4. Barbero-Villares A, Mendoza Jiménez-Ridruejo J, Taxonera C, López-Sanromán A, Pajares R, Bermejo F, Pérez-Calle JL, et al. Evaluation of liver fibrosis by transient elastography (Fibroscan®) in patients with inflammatory bowel disease treated with methotrexate: a multicentric trial. Scand J Gastroenterol 2012;47:575-579.

5. Borren NZ, Luther J, Colizzo FP, Garber JG, Khalili H, Ananthakrishnan AN. Low-dose Methotrexate has Similar Outcomes to High-dose Methotrexate in Combination with Anti-TNF Therapy in Inflammatory Bowel Diseases. Aliment Pharmacol Ther 2019;13:990-995.
6. Boyle B, Mackner L, Ross C, Moses J, Kumar S, Crandall W. A single-center experience with methotrexate after thiopurine therapy in pediatric Crohn disease. J Pediatr Gastroenterol Nutr 2010;51:714-717.

7. Carbonnel F, Colombel JF, Filippi J, Katsanos KH, Peyrin-Biroulet L, Allez M, Nachury M, et al. Methotrexate Is Not Superior to Placebo for Inducing Steroid-Free Remission, but Induces Steroid-Free Clinical Remission in a Larger Proportion of Patients With Ulcerative Colitis. Gastroenterology 2016;150:380-388.e384.

8. Chande N, Abdelgadir I, Gregor J. The safety and tolerability of methotrexate for treating patients with Crohn's disease. J Clin Gastroenterol 2011;45:599-601.

9. Charpignon C, Beau P. Methotrexate as single therapy in Crohn's disease: is its long-term efficacy limited? Gastroenterol Clin Biol 2008;32:153-157.

10. Chong RY, Hanauer SB, Cohen RD. Efficacy of parenteral methotrexate in refractory Crohn's disease. Aliment Pharmacol Ther 2001;15:35-44.

11. Colman RJ, Rubin DT. Optimal doses of methotrexate combined with anti-TNF therapy to maintain clinical remission in inflammatory bowel disease. J Crohns Colitis 2015;9:312-317.

12. Cummings JR, Herrlinger KR, Travis SP, Gorard DA, McIntyre AS, Jewell DP. Oral methotrexate in ulcerative colitis. Aliment Pharmacol Ther 2005;21:385-389.

13. Din S, Dahele A, Fennel J, Aitken S, Shand AG, Arnott ID, Satsangi J. Use of methotrexate in refractory Crohn's disease: the Edinburgh experience. Inflamm Bowel Dis 2008;14:756-762.

14. Domènech E, Mañosa M, Navarro M, Masnou H, Garcia-Planella E, Zabana Y, Cabré E, et al. Long-term methotrexate for Crohn's disease: safety and efficacy in clinical practice. J Clin Gastroenterol 2008;42:395-399.

15. Dupont-Lucas C, Grandjean-Blanchet C, Leduc B, Tripcovici M, Larocque C, Gervais F, Jantchou P, et al. Prevalence and Risk Factors for Symptoms of Methotrexate Intolerance in Pediatric Inflammatory Bowel Disease. Inflamm Bowel Dis 2017;23:298-303.

16. Egan LJ, Sandborn WJ, Tremaine WJ, Leighton JA, Mays DC, Pike MG, Zinsmeister AR, et al. A randomized dose-response and pharmacokinetic study of methotrexate for refractory inflammatory Crohn's disease and ulcerative colitis. Aliment Pharmacol Ther 1999;13:1597-1604.

17. Feagan BG, Fedorak RN, Irvine EJ, Wild G, Sutherland L, Steinhart AH, Greenberg GR, et al. A comparison of methotrexate with placebo for the maintenance of remission in Crohn's disease. North American Crohn's Study Group Investigators. N Engl J Med 2000;342:1627-1632.

18. Feagan BG, McDonald JW, Panaccione R, Enns RA, Bernstein CN, Ponich TP, Bourdages R, et al. Methotrexate in combination with infliximab is no more effective than infliximab alone in patients with Crohn's disease. Gastroenterology 2014;146:681-688.e681.

19. Feagan BG, Rochon J, Fedorak RN, Irvine EJ, Wild G, Sutherland L, Steinhart AH, et al. Methotrexate for the treatment of Crohn's disease. The North American Crohn's Study Group Investigators. N Engl J Med 1995;332:292-297.

20. Fournier MR, Klein J, Minuk GY, Bernstein CN. Changes in liver biochemistry during methotrexate use for inflammatory bowel disease. Am J Gastroenterol 2010;105:1620-1626.

21. Fraser AG, Morton D, McGovern D, Travis S, Jewell DP. The efficacy of methotrexate for maintaining remission in inflammatory bowel disease. Aliment Pharmacol Ther 2002;16:693-697.

22. González-Lama Y, Taxonera C, López-Sanromán A, Pérez-Calle JL, Bermejo F, Pajares R, McNicholl AG, et al. Methotrexate in inflammatory bowel disease: a multicenter retrospective study focused on long-term efficacy and safety. The Madrid experience. Case Rep Rheumatol 2012;24:1086-1091.

23. Haisma SM, Lijftogt T, Kindermann A, Damen G, de Ridder L, Escher JC, Mearin ML, et al. Methotrexate for maintaining remission in paediatric Crohn's patients with prior failure or intolerance to thiopurines: a multicenter cohort study. J Crohns Colitis 2015;9:305-311.

24. Hausmann J, Zabel K, Herrmann E, Schröder O. Methotrexate for maintenance of remission in chronic active Crohn's disease: long-term single-center experience and meta-analysis of observational studies. Inflamm Bowel Dis 2010;16:1195-1202.

25. Hayee BH, Harris AW. Methotrexate for Crohn's disease: experience in a district general hospital. Eur J Gastroenterol Hepatol 2005;17:893-898.

26. Herfarth H, Barnes EL, Valentine JF, Hanson J, Higgins PDR, Isaacs KL, Jackson S, et al. Methotrexate Is Not Superior to Placebo in Maintaining Steroid-Free Response or Remission in Ulcerative Colitis. Gastroenterology 2018;155:1098-1108.e1099.

27. Herrlinger KR, Cummings JR, Barnardo MC, Schwab M, Ahmad T, Jewell DP. The pharmacogenetics of methotrexate in inflammatory bowel disease. Pharmacogenet Genomics 2005;15:705-711.

28. Hojsak I, Mišak Z, Jadrešin O, Močić Pavić A, Kolaček S. Methotrexate is an efficient therapeutic alternative in children with thiopurine-resistant Crohn's disease. Scand J Gastroenterol 2015;50:1208-1213.

29. Huang Z, Chao K, Li M, Zhi M, Tang J, Hu P, Gao X. Methotrexate for Refractory Crohn's Disease Compared with Thiopurines: A Retrospective Non-head-to-head Controlled Study. Inflamm Bowel Dis 2017;23:440-447.

30. Kopylov U, Katsanos KH, van der Woude CJ, Karmiris K, Hernandez V, Odes S, Papamichael K, et al. European experience with methotrexate treatment in Crohn's disease: a multicenter retrospective analysis. Eur J Gastroenterol Hepatol 2016;28:802-806.

31. Kozarek RA, Patterson DJ, Gelfand MD, Botoman VA, Ball TJ, Wilske KR. Methotrexate induces clinical and histologic remission in patients with refractory inflammatory bowel disease. Ann Intern Med 1989;110:353-356.

32. Laharie D, Zerbib F, Adhoute X, Boué-Lahorgue X, Foucher J, Castéra L, Rullier A, et al. Diagnosis of liver fibrosis by transient elastography (FibroScan) and non-invasive methods in Crohn's disease patients treated with methotrexate. Aliment Pharmacol Ther 2006;23:1621-1628.

33. Lémann M, Chamiot-Prieur C, Mesnard B, Halphen M, Messing B, Rambaud JC, Gendre JP, et al. Methotrexate for the treatment of refractory Crohn's disease. Aliment Pharmacol Ther 1996;10:309-314.

34. Lémann M, Zenjari T, Bouhnik Y, Cosnes J, Mesnard B, Rambaud JC, Modigliani R, et al. Methotrexate in Crohn's disease: long-term efficacy and toxicity. Am J Gastroenterol 2000;95:1730-1734.

35. Llaó J, Masnou H, Romero C, Bargalló A, Gely C, Mañosa M, Gordillo J, et al. Noninvasive assessment of liver fibrosis in Crohn's disease patients exposed to methotrexate. Eur J Gastroenterol Hepatol 2020.

36. Mañosa M, García V, Castro L, García-Bosch O, Chaparro M, Barreiro-de Acosta M, Carpio D, et al. Methotrexate in ulcerative colitis: a Spanish multicentric study on clinical use and efficacy. J Crohns Colitis 2011;5:397-401.

37. Maté-Jiménez J, Hermida C, Cantero-Perona J, Moreno-Otero R. 6-mercaptopurine or methotrexate added to prednisone induces and maintains remission in steroid-dependent inflammatory bowel disease. Eur J Gastroenterol Hepatol 2000;12:1227-1233.

38. Meijer B, Mulder CJJ, Bouma G, Ponsioen CY, van der Woude CJ, van der Meulen AE, Wintjens DSJ, et al. Methotrexate and Thioguanine Rescue Therapy for Conventional Thiopurine Failing Ulcerative Colitis Patients: A Multi-center Database Study on Tolerability and Effectiveness. Inflamm Bowel Dis 2018;24:1558-1565.

39. Nathan DM, Iser JH, Gibson PR. A single center experience of methotrexate in the treatment of Crohn's disease and ulcerative colitis: a case for subcutaneous administration. J Gastroenterol Hepatol 2008;23:954-958.

40. Oren R, Arber N, Odes S, Moshkowitz M, Keter D, Pomeranz I, Ron Y, et al. Methotrexate in chronic active ulcerative colitis: a double-blind, randomized, Israeli multicenter trial. Gastroenterology 1996;110:1416-1421.

41. Pedroto I, Lago P, Vasudevan A. Thiopurines vs methotrexate: Comparing tolerability and discontinuation rates in the treatment of inflammatory bowel disease. Rev Esp Enferm Dig 2020;52:1174-1184.

42. Rouiller-Braunschweig C, Fournier N, Pittet V, Dudler J, Michetti P. Efficacy, Safety and Mucosal Healing of Methotrexate in a Large Longitudinal Cohort of Inflammatory Bowel Disease Patients. Digestion 2017;96:220-227.

43. Roumeguère P, Bouchard D, Pigot F, Castinel A, Juguet F, Gaye D, Capdepont M, et al. Combined approach with infliximab, surgery, and methotrexate in severe fistulizing anoperineal Crohn's disease: results from a prospective study. Inflamm Bowel Dis 2011;17:69-76.

44. Saibeni S, Bollani S, Losco A, Michielan A, Sostegni R, Devani M, Lupinacci G, et al. The use of methotrexate for treatment of inflammatory bowel disease in clinical practice. Dig Liver Dis 2012;44:123-127.

45. Schröder T, Schmidt KJ, Olsen V, Möller S, Mackenroth T, Sina C, Lehnert H, et al. Liver steatosis is a risk factor for hepatotoxicity in patients with inflammatory bowel disease under immunosuppressive treatment. Eur J Gastroenterol Hepatol 2015;27:698-704.

46. Seinen ML, Ponsioen CY, de Boer NK, Oldenburg B, Bouma G, Mulder CJ, van Bodegraven AA. Sustained clinical benefit and tolerability of methotrexate monotherapy after thiopurine therapy in patients with Crohn's disease. Clin Gastroenterol Hepatol 2013;11:667-672.

47. Suares NC, Hamlin PJ, Greer DP, Warren L, Clark T, Ford AC. Efficacy and tolerability of methotrexate therapy for refractory Crohn's disease: a large single-centre experience. Aliment Pharmacol Ther 2012;35:284-291.

48. Sunseri W, Hyams JS, Lerer T, Mack DR, Griffiths AM, Otley AR, Rosh JR, et al. Retrospective cohort study of methotrexate use in the treatment of pediatric Crohn's disease. Inflamm Bowel Dis 2014;20:1341-1345.

49. Te HS, Schiano TD, Kuan SF, Hanauer SB, Conjeevaram HS, Baker AL. Hepatic effects of long-term methotrexate use in the treatment of inflammatory bowel disease. Am J Gastroenterol 2000;95:3150-3156.

50. Turner D, Doveh E, Cohen A, Wilson ML, Grossman AB, Rosh JR, Lu Y, et al. Efficacy of oral methotrexate in paediatric Crohn's disease: a multicentre propensity score study. Gut 2015;64:1898-1904.

51. Turner D, Grossman AB, Rosh J, Kugathasan S, Gilman AR, Baldassano R, Griffiths AM. Methotrexate following unsuccessful thiopurine therapy in pediatric Crohn's disease. Am J Gastroenterol 2007;102:2804-2812; quiz 2803, 2813.

52. Uhlen S, Belbouab R, Narebski K, Goulet O, Schmitz J, Cézard JP, Turck D, et al. Efficacy of methotrexate in pediatric Crohn's disease: a French multicenter study. Inflamm Bowel Dis 2006;12:1053-1057.

53. Vandeputte L, D'Haens G, Baert F, Rutgeerts P. Methotrexate in refractory Crohn's disease. Inflamm Bowel Dis 1999;5:11-15.

54. Vasudevan A. Higher Mucosal Healing with Tumor Necrosis Factor Inhibitors in Combination with Thiopurines Compared to Methotrexate in Crohn's Disease. Biomed Res Int 2019;64:1622-1631.

55. Wahed M, Louis-Auguste JR, Baxter LM, Limdi JK, McCartney SA, Lindsay JO, Bloom SL. Efficacy of methotrexate in Crohn's disease and ulcerative colitis patients unresponsive or intolerant to azathioprine /mercaptopurine. Aliment Pharmacol Ther 2009;30:614-620.

56. Weiss B, Lerner A, Shapiro R, Broide E, Levine A, Fradkin A, Bujanover Y. Methotrexate treatment in pediatric Crohn disease patients intolerant or resistant to purine analogues. J Pediatr Gastroenterol Nutr 2009;48:526-530.

57. Willot S, Noble A, Deslandres C. Methotrexate in the treatment of inflammatory bowel disease: an 8-year retrospective study in a Canadian pediatric IBD center. Inflamm Bowel Dis 2011;17:2521-2526.

58. Zelinkova Z, Bultman E, Vogelaar L, Bouziane C, Kuipers EJ, van der Woude CJ. Sex-dimorphic adverse drug reactions to immune suppressive agents in inflammatory bowel disease. World J Gastroenterol 2012;18:6967-6973.

**Included articles of rheumatoid arthritis:**

1. Ali AA, Iqbal MP, Hussain MA, Mehboobali N, Beg JA, Rahbar MH. Methotrexate in rheumatoid arthritis: a 2 year experience at a university hospital in Pakistan. J Pak Med Assoc 1998;48:3-6.

2. Alten RE, Zerbini C, Jeka S, Irazoque F, Khatib F, Emery P, Bertasso A, et al. Efficacy and safety of pamapimod in patients with active rheumatoid arthritis receiving stable methotrexate therapy. Ann Rheum Dis 2010;69:364-367.

3. Amital H, Arnson Y, Chodick G, Shalev V. Hepatotoxicity rates do not differ in patients with rheumatoid arthritis and psoriasis treated with methotrexate. Rheumatology (Oxford) 2009;48:1107-1110.

4. Aponte J, Petrelli M. Histopathologic findings in the liver of rheumatoid arthritis patients treated with long-term bolus methotrexate. Arthritis Rheum 1988;31:1457-1464.

5. Baraldo M, Ferraccioli G, Pea F, Gremese E, Furlanut M. Cyclosporine A pharmacokinetics in rheumatoid arthritis patients after 6 months of methotrexate therapy. Pharmacol Res 1999;40:483-486.

6. Berkun Y, Abou Atta I, Rubinow A, Orbach H, Levartovsky D, Aamar S, Arbel O, et al. 2756GG genotype of methionine synthase reductase gene is more prevalent in rheumatoid arthritis patients treated with methotrexate and is associated with methotrexate-induced nodulosis. J Rheumatol 2007;34:1664-1669.

7. Beyeler C, Reichen J, Thomann SR, Lauterburg BH, Gerber NJ. Quantitative liver function in patients with rheumatoid arthritis treated with low-dose methotrexate: a longitudinal study. Br J Rheumatol 1997;36:338-344.

8. Bird P, Griffiths H, Tymms K, Nicholls D, Roberts L, Arnold M, Burnet S, et al. The SMILE study -- safety of methotrexate in combination with leflunomide in rheumatoid arthritis. J Rheumatol 2013;40:228-235.

9. Bjorkman DJ, Hammond EH, Lee RG, Clegg DO, Tolman KG. Hepatic ultrastructure after methotrexate therapy for rheumatoid arthritis. Arthritis Rheum 1988;31:1465-1472.

10. Boers M, Verhoeven AC, Markusse HM, van de Laar MA, Westhovens R, van Denderen JC, van Zeben D, et al. Randomised comparison of combined step-down prednisolone, methotrexate and sulphasalazine with sulphasalazine alone in early rheumatoid arthritis. Lancet 1997;350:309-318.

11. Bohanec Grabar P, Logar D, Lestan B, Dolzan V. Genetic determinants of methotrexate toxicity in rheumatoid arthritis patients: a study of polymorphisms affecting methotrexate transport and folate metabolism. Eur J Clin Pharmacol 2008;64:1057-1068.

12. Braun J, Kastner P, Flaxenberg P, Wahrisch J, Hanke P, Demary W, von Hinuber U, et al. Comparison of the clinical efficacy and safety of subcutaneous versus oral administration of methotrexate in patients with active rheumatoid arthritis: results of a six-month, multicenter, randomized, double-blind, controlled, phase IV trial. Arthritis Rheum 2008;58:73-81.

13. Breedveld FC, Weisman MH, Kavanaugh AF, Cohen SB, Pavelka K, van Vollenhoven R, Sharp J, et al. The PREMIER study: A multicenter, randomized, double-blind clinical trial of combination therapy with adalimumab plus methotrexate versus methotrexate alone or adalimumab alone in patients with early, aggressive rheumatoid arthritis who had not had previous methotrexate treatment. Arthritis Rheum 2006;54:26-37.

14. Burmester GR, Blanco R, Charles-Schoeman C, Wollenhaupt J, Zerbini C, Benda B, Gruben D, et al. Tofacitinib (CP-690,550) in combination with methotrexate in patients with active rheumatoid arthritis with an inadequate response to tumour necrosis factor inhibitors: a randomised phase 3 trial. Lancet 2013;381:451-460.

15. Cáliz R, del Amo J, Balsa A, Blanco F, Silva L, Sanmarti R, Martínez FG, et al. The C677T polymorphism in the MTHFR gene is associated with the toxicity of methotrexate in a Spanish rheumatoid arthritis population. Scand J Rheumatol 2012;41:10-14.

16. Chen L, Qi H, Jiang D, Wang R, Chen A, Yan Z, Xiao J. The new use of an ancient remedy: a double-blinded randomized study on the treatment of rheumatoid arthritis. Am J Chin Med 2013;41:263-280.

17. Chen XX, Dai Q, Huang AB, Wu HX, Zhao DB, Li XF, Hu SX, et al. A multicenter, randomized, double-blind clinical trial of combination therapy with Anbainuo, a novel recombinant human TNFRII:Fc fusion protein, plus methotrexate versus methotrexate alone or Anbainuo alone in Chinese patients with moderate to severe rheumatoid arthritis. Clin Rheumatol 2013;32:99-108.

18. Chen Z, Li XP, Li ZJ, Xu L, Li XM. Reduced hepatotoxicity by total glucosides of paeony in combination treatment with leflunomide and methotrexate for patients with active rheumatoid arthritis. Int Immunopharmacol 2013;15:474-477.

19. Choe JY, Lee H, Jung HY, Park SH, Bae SC, Kim SK. Methylenetetrahydrofolate reductase polymorphisms, C677T and A1298C, are associated with methotrexate-related toxicities in Korean patients with rheumatoid arthritis. Rheumatol Int 2012;32:1837-1842.

20. Cohen S, Cannon GW, Schiff M, Weaver A, Fox R, Olsen N, Furst D, et al. Two-year, blinded, randomized, controlled trial of treatment of active rheumatoid arthritis with leflunomide compared with methotrexate. Utilization of Leflunomide in the Treatment of Rheumatoid Arthritis Trial Investigator Group. Arthritis Rheum 2001;44:1984-1992.

21. Cohen SB, Cheng TT, Chindalore V, Damjanov N, Burgos-Vargas R, Delora P, Zimany K, et al. Evaluation of the efficacy and safety of pamapimod, a p38 MAP kinase inhibitor, in a double-blind, methotrexate-controlled study of patients with active rheumatoid arthritis. Arthritis Rheum 2009;60:335-344.

22. Coleiro B, Mallia C. Toxicity profile of methotrexate in rheumatoid arthritis. A preliminary survey. Adv Exp Med Biol 1999;455:359-365.

23. Davis LA, Polk B, Mann A, Wolff RK, Kerr GS, Reimold AM, Cannon GW, et al. Folic acid pathway single nucleotide polymorphisms associated with methotrexate significant adverse events in United States veterans with rheumatoid arthritis. Clin Exp Rheumatol 2014;32:324-332.

24. de Rotte MC, de Jong PH, Pluijm SM, Calasan MB, Barendregt PJ, van Zeben D, van der Lubbe PA, et al. Association of low baseline levels of erythrocyte folate with treatment nonresponse at three months in rheumatoid arthritis patients receiving methotrexate. Arthritis Rheum 2013;65:2803-2813.

25. de Thurah A, Nørgaard M, Johansen MB, Stengaard-Pedersen K. Methotrexate compliance among patients with rheumatoid arthritis: the influence of disease activity, disease duration, and co-morbidity in a 10-year longitudinal study. Scand J Rheumatol 2010;39:197-205.

26. Dervieux T, Wessels JA, van der Straaten T, Penrod N, Moore JH, Guchelaar HJ, Kremer JM. Gene-gene interactions in folate and adenosine biosynthesis pathways affect methotrexate efficacy and tolerability in rheumatoid arthritis. Pharmacogenet Genomics 2009;19:935-944.

27. Dhir V, Singla M, Gupta N, Goyal P, Sagar V, Sharma A, Khanna S, et al. Randomized controlled trial comparing 2 different starting doses of methotrexate in rheumatoid arthritis. Clin Ther 2014;36:1005-1015.

28. Ding CZ, Yao Y, Feng XB, Fang Y, Zhao C, Wang Y. Clinical analysis of chinese patients with rheumatoid arthritis treated with leflunomide and methotrexate combined with different dosages of glucocorticoid. Curr Ther Res Clin Exp 2012;73:123-133.

29. Dirven L, Klarenbeek NB, van den Broek M, van Groenendael JH, de Sonnaville PB, Kerstens PJ, Huizinga TW, et al. Risk of alanine transferase (ALT) elevation in patients with rheumatoid arthritis treated with methotrexate in a DAS-steered strategy. Clin Rheumatol 2013;32:585-590.

30. Dougados M, Combe B, Cantagrel A, Goupille P, Olive P, Schattenkirchner M, Meusser S, et al. Combination therapy in early rheumatoid arthritis: a randomised, controlled, double blind 52 week clinical trial of sulphasalazine and methotrexate compared with the single components. Ann Rheum Dis 1999;58:220-225.

31. Drosos AA, Psychos D, Andonopoulos AP, Stefanaki-Nikou S, Tsianos EB, Moutsopoulos HM. Methotrexate therapy in rheumatoid arthritis. A two year prospective follow-up. Clin Rheumatol 1990;9:333-341.

32. Drozdzik M, Rudas T, Pawlik A, Gornik W, Kurzawski M, Herczynska M. Reduced folate carrier-1 80G>A polymorphism affects methotrexate treatment outcome in rheumatoid arthritis. Pharmacogenomics J 2007;7:404-407.

33. Dubey L, Chatterjee S, Ghosh A. Hepatic and hematological adverse effects of long-term low-dose methotrexate therapy in rheumatoid arthritis: An observational study. Indian J Pharmacol 2016;48:591-594.

34. Emery P, Breedveld FC, Lemmel EM, Kaltwasser JP, Dawes PT, Gömör B, Van Den Bosch F, et al. A comparison of the efficacy and safety of leflunomide and methotrexate for the treatment of rheumatoid arthritis. Rheumatology (Oxford) 2000;39:655-665.

35. Emery P, Fleischmann RM, Moreland LW, Hsia EC, Strusberg I, Durez P, Nash P, et al. Golimumab, a human anti-tumor necrosis factor alpha monoclonal antibody, injected subcutaneously every four weeks in methotrexate-naive patients with active rheumatoid arthritis: twenty-four-week results of a phase III, multicenter, randomized, double-blind, placebo-controlled study of golimumab before methotrexate as first-line therapy for early-onset rheumatoid arthritis. Arthritis Rheum 2009;60:2272-2283.

36. Erickson AR, Reddy V, Vogelgesang SA, West SG. Usefulness of the American College of Rheumatology recommendations for liver biopsy in methotrexate-treated rheumatoid arthritis patients. Arthritis Rheum 1995;38:1115-1119.

37. Erre GL, Cadoni ML, Meloni P, Castagna F, Mangoni AA, Piga M, Passiu G, et al. Methotrexate therapy is not associated with increased liver stiffness and significant liver fibrosis in rheumatoid arthritis patients: A cross-sectional controlled study with real-time two-dimensional shear wave elastography. Eur J Intern Med 2019;69:57-63.

38. Fathi NH, Mitros F, Hoffman J, Straniero N, Labreque D, Koehnke R, Furst DE. Longitudinal measurement of methotrexate liver concentrations does not correlate with liver damage, clinical efficacy, or toxicity during a 3.5 year double blind study in rheumatoid arthritis. J Rheumatol 2002;29:2092-2098.

39. Fleischmann R, Pangan AL, Song IH, Mysler E, Bessette L, Peterfy C, Durez P, et al. Upadacitinib Versus Placebo or Adalimumab in Patients With Rheumatoid Arthritis and an Inadequate Response to Methotrexate: Results of a Phase III, Double-Blind, Randomized Controlled Trial. 2019;71:1788-1800.

40. García DS, Saturansky EI, Poncino D, Martínez-Artola Y, Rosenberg S, Abritta G, Ascimani-Peña C, et al. "Hepatic toxicity by methotrexate with weekly single doses associated with folic acid in rheumatoid and psoriatic arthritis. What is its real frequency?". Ann Hepatol 2019;18:765-769.

41. Genovese MC, Fleischmann R, Kivitz AJ, Rell-Bakalarska M, Martincova R, Fiore S, Rohane P, et al. Sarilumab Plus Methotrexate in Patients With Active Rheumatoid Arthritis and Inadequate Response to Methotrexate: Results of a Phase III Study. Arthritis Rheumatol 2015;67:1424-1437.

42. Genovese MC, Smolen JS, Weinblatt ME, Burmester GR, Meerwein S, Camp HS, Wang L, et al. Efficacy and Safety of ABT-494, a Selective JAK-1 Inhibitor, in a Phase IIb Study in Patients With Rheumatoid Arthritis and an Inadequate Response to Methotrexate. Arthritis Rheumatol 2016;68:2857-2866.

43. Genovese MC, van Adelsberg J, Fan C, Graham NMH, van Hoogstraten H, Parrino J, Mangan EK, et al. Two years of sarilumab in patients with rheumatoid arthritis and an inadequate response to MTX: safety, efficacy and radiographic outcomes. Rheumatology (Oxford) 2018;57:1423-1431.

44. Genovese MC, van Vollenhoven RF, Pacheco-Tena C, Zhang Y, Kinnman N. VX-509 (Decernotinib), an Oral Selective JAK-3 Inhibitor, in Combination With Methotrexate in Patients With Rheumatoid Arthritis. Arthritis Rheumatol 2016;68:46-55.

45. Gerards AH, Landewe RB, Prins AP, Bruyn GA, Goei The HS, Laan RF, Dijkmans BA. Cyclosporin A monotherapy versus cyclosporin A and methotrexate combination therapy in patients with early rheumatoid arthritis: a double blind randomised placebo controlled trial. Ann Rheum Dis 2003;62:291-296.

46. Gilani ST, Khan DA, Khan FA, Ahmed M. Adverse effects of low dose methotrexate in rheumatoid arthritis patients. J Coll Physicians Surg Pak 2012;22:101-104.

47. Graham LD, Myones BL, Rivas-Chacon RF, Pachman LM. Morbidity associated with long-term methotrexate therapy in juvenile rheumatoid arthritis. J Pediatr 1992;120:468-473.

48. Haagsma CJ, van Riel PL, de Jong AJ, van de Putte LB. Combination of sulphasalazine and methotrexate versus the single components in early rheumatoid arthritis: a randomized, controlled, double-blind, 52 week clinical trial. Br J Rheumatol 1997;36:1082-1088.

49. Hara M, Ishiguro N, Katayama K, Kondo M, Sumida T, Mimori T, Soen S, et al. Safety and efficacy of combination therapy of iguratimod with methotrexate for patients with active rheumatoid arthritis with an inadequate response to methotrexate: an open-label extension of a randomized, double-blind, placebo-controlled trial. Mod Rheumatol 2014;24:410-418.

50. Harris E. Using subcutaneous methotrexate to prolong duration of methotrexate therapy in rheumatoid arthritis. Int J Rheumatol 2018;5:85-91.

51. Hayashi T, Ito S, Goto D, Matsumoto I, Sumida T. Elevated level of serum cystatin-C concentration is a useful predictor for myelosuppression induced by methotrexate for treatment of rheumatoid arthritis. Mod Rheumatol 2010;20:548-555.

52. He L, Boughrara W, Benzaoui A, Aberkane M, Moghtit FZ, Dorgham S, Lardjam-Hetraf AS, et al. No correlation between MTHFR c.677 C > T, MTHFR c.1298 A > C, and ABCB1 c.3435 C > T polymorphisms and methotrexate therapeutic outcome of rheumatoid arthritis in West Algerian population. Medicine (Baltimore) 2017;66:505-513.

53. Hirshberg B, Muszkat M, Schlesinger O, Rubinow A. Safety of low dose methotrexate in elderly patients with rheumatoid arthritis. Postgrad Med J 2000;76:787-789.

54. Hoekstra M, van Ede AE, Haagsma CJ, van de Laar MA, Huizinga TW, Kruijsen MW, Laan RF. Factors associated with toxicity, final dose, and efficacy of methotrexate in patients with rheumatoid arthritis. Ann Rheum Dis 2003;62:423-426.

55. Hoffmeister RT. Methotrexate therapy in rheumatoid arthritis: 15 years experience. Am J Med 1983;75:69-73.

56. Hu D, Bao C, Chen S, Gu J, Li Z, Sun L, Han X, et al. A comparison study of a recombinant tumor necrosis factor receptor:Fc fusion protein (rhTNFR:Fc) and methotrexate in treatment of patients with active rheumatoid arthritis in China. Rheumatol Int 2009;29:297-303.

57. Hua L, Du H, Ying M, Wu H, Fan J, Shi X. Efficacy and safety of low-dose glucocorticoids combined with methotrexate and hydroxychloroquine in the treatment of early rheumatoid arthritis: A single-center, randomized, double-blind clinical trial. Medicine (Baltimore) 2020;99:e20824.

58. Huang RY, Pan HD, Wu JQ, Zhou H, Li ZG, Qiu P, Zhou YY, et al. Comparison of combination therapy with methotrexate and sinomenine or leflunomide for active rheumatoid arthritis: A randomized controlled clinical trial. Int J Rheum Dis 2019;57:403-410.

59. Hughes LB, Beasley TM, Patel H, Tiwari HK, Morgan SL, Baggott JE, Saag KG, et al. Racial or ethnic differences in allele frequencies of single-nucleotide polymorphisms in the methylenetetrahydrofolate reductase gene and their influence on response to methotrexate in rheumatoid arthritis. Ann Rheum Dis 2006;65:1213-1218.

60. Huizinga TW, Fleischmann RM, Jasson M, Radin AR, van Adelsberg J, Fiore S, Huang X, et al. Sarilumab, a fully human monoclonal antibody against IL-6Rα in patients with rheumatoid arthritis and an inadequate response to methotrexate: efficacy and safety results from the randomised SARIL-RA-MOBILITY Part A trial. Ann Rheum Dis 2014;73:1626-1634.

61. Humphreys JH, Warner A, Costello R. Quantifying the hepatotoxic risk of alcohol consumption in patients with rheumatoid arthritis taking methotrexate. 2017;76:1509-1514.

62. Ikeda K, Watanabe K, Hirai T, Tanji K, Miyashita T, Nakajima S, Uomori K, et al. Mizoribine Synchronized Methotrexate Therapy should be Considered when Treating Rheumatoid Arthritis Patients with an Inadequate Response to Various Combination Therapies. Intern Med 2017;56:1147-1152.

63. Ishaq M, Muhammad JS, Hameed K, Mirza AI. Leflunomide or methotrexate? Comparison of clinical efficacy and safety in low socio-economic rheumatoid arthritis patients. Mod Rheumatol 2011;21:375-380.

64. Jones G, Sebba A, Gu J, Lowenstein MB, Calvo A, Gomez-Reino JJ, Siri DA, et al. Comparison of tocilizumab monotherapy versus methotrexate monotherapy in patients with moderate to severe rheumatoid arthritis: the AMBITION study. Ann Rheum Dis 2010;69:88-96.

65. Kameyama S, Kase Y, Kurihara S, Yoshida F, Noda M, Iiduka T, Horiguchi M, et al. Influence of High-Dose Folic Acid on Methotrexate Efficacies and Safety in Japanese Rheumatoid Arthritis Patients. Drug Res (Stuttg) 2017;67:705-709.

66. Karlsson Sundbaum J, Eriksson N, Hallberg P, Lehto N, Wadelius M, Baecklund E. Methotrexate treatment in rheumatoid arthritis and elevated liver enzymes: A long-term follow-up of predictors, surveillance, and outcome in clinical practice. Int J Rheum Dis 2019;22:1226-1232.

67. Kent PD, Luthra HS, Michet C, Jr. Risk factors for methotrexate-induced abnormal laboratory monitoring results in patients with rheumatoid arthritis. J Rheumatol 2004;31:1727-1731.

68. Keystone EC, Genovese MC, Klareskog L, Hsia EC, Hall ST, Miranda PC, Pazdur J, et al. Golimumab, a human antibody to tumour necrosis factor {alpha} given by monthly subcutaneous injections, in active rheumatoid arthritis despite methotrexate therapy: the GO-FORWARD Study. Ann Rheum Dis 2009;68:789-796.

69. Keystone EC, Taylor PC, Drescher E, Schlichting DE, Beattie SD, Berclaz PY, Lee CH, et al. Safety and efficacy of baricitinib at 24 weeks in patients with rheumatoid arthritis who have had an inadequate response to methotrexate. Intern Med J 2015;74:333-340.

70. Khan H, Hakamata J, Hashiguchi M, Kaneko Y, Yamaoka K, Shimizu M, Maruyama J, et al. Risk factors for abnormal hepatic enzyme elevation by methotrexate treatment in patients with rheumatoid arthritis: A hospital based-cohort study. Biomed Res Int 2018;28:611-620.

71. Kim SK, Jun JB, El-Sohemy A, Bae SC. Cost-effectiveness analysis of MTHFR polymorphism screening by polymerase chain reaction in Korean patients with rheumatoid arthritis receiving methotrexate. J Rheumatol 2006;33:1266-1274.

72. Kim TY, Kim JY, Sohn JH, Lee HS, Bang SY, Kim Y, Kim MY, et al. Assessment of Substantial Liver Fibrosis by Real-time Shear Wave Elastography in Methotrexate-Treated Patients With Rheumatoid Arthritis. J Ultrasound Med 2015;34:1621-1630.

73. Kivitz AJ, Gutierrez-Ureña SR, Poiley J, Genovese MC, Kristy R, Shay K, Wang X, et al. Peficitinib, a JAK Inhibitor, in the Treatment of Moderate-to-Severe Rheumatoid Arthritis in Patients With an Inadequate Response to Methotrexate. Arthritis Rheumatol 2017;69:709-719.

74. Klareskog L, van der Heijde D, de Jager JP, Gough A, Kalden J, Malaise M, Martin Mola E, et al. Therapeutic effect of the combination of etanercept and methotrexate compared with each treatment alone in patients with rheumatoid arthritis: double-blind randomised controlled trial. Lancet 2004;363:675-681.

75. Kremer J, Genovese M, Cannon GW, Caldwell J, Cush J, Furst DE, Luggen M, et al. Combination leflunomide and methotrexate (MTX) therapy for patients with active rheumatoid arthritis failing MTX monotherapy: open-label extension of a randomized, double-blind, placebo controlled trial. J Rheumatol 2004;31:1521-1531.

76. Kremer JM, Cohen S, Wilkinson BE, Connell CA, French JL, Gomez-Reino J, Gruben D, et al. A phase IIb dose-ranging study of the oral JAK inhibitor tofacitinib (CP-690,550) versus placebo in combination with background methotrexate in patients with active rheumatoid arthritis and an inadequate response to methotrexate alone. Arthritis Rheum 2012;64:970-981.

77. Kremer JM, Genovese MC, Cannon GW, Caldwell JR, Cush JJ, Furst DE, Luggen ME, et al. Concomitant leflunomide therapy in patients with active rheumatoid arthritis despite stable doses of methotrexate. A randomized, double-blind, placebo-controlled trial. Ann Intern Med 2002;137:726-733.

78. Kremer JM, Lee JK. The safety and efficacy of the use of methotrexate in long-term therapy for rheumatoid arthritis. Arthritis Rheum 1986;29:822-831.

79. Kremer JM, Lee RG, Tolman KG. Liver histology in rheumatoid arthritis patients receiving long-term methotrexate therapy. A prospective study with baseline and sequential biopsy samples. Arthritis Rheum 1989;32:121-127.

80. Kremer JM, Phelps CT. Long-term prospective study of the use of methotrexate in the treatment of rheumatoid arthritis. Update after a mean of 90 months. Arthritis Rheum 1992;35:138-145.

81. Lee EB, Fleischmann R, Hall S, Wilkinson B, Bradley JD, Gruben D, Koncz T, et al. Tofacitinib versus methotrexate in rheumatoid arthritis. Arthritis Rheumatol 2014;370:2377-2386.

82. Lee SS, Park YW, Park JJ, Kang YM, Nam EJ, Kim SI, Lee JH, et al. Combination treatment with leflunomide and methotrexate for patients with active rheumatoid arthritis. Scand J Rheumatol 2009;38:11-14.

83. Leonard PA, Clegg DO, Carson CC, Cannon GW, Egger MJ, Ward JR. Low dose pulse methotrexate in rheumatoid arthritis: an 8-year experience with hepatotoxicity. Clin Rheumatol 1987;6:575-582.

84. Lerndal T, Svensson B. A clinical study of CPH 82 vs methotrexate in early rheumatoid arthritis. Rheumatology (Oxford) 2000;39:316-320.

85. Lertnawapan R, Chonprasertsuk S. Association between cumulative methotrexate dose, non-invasive scoring system and hepatic fibrosis detected by Fibroscan in rheumatoid arthritis patients receiving methotrexate. 2019;22:214-221.

86. Lie E, van der Heijde D, Uhlig T, Heiberg MS, Koldingsnes W, Rødevand E, Kaufmann C, et al. Effectiveness and retention rates of methotrexate in psoriatic arthritis in comparison with methotrexate-treated patients with rheumatoid arthritis. Ann Rheum Dis 2010;69:671-676.

87. Lima A, Bernardes M, Azevedo R, Monteiro J, Sousa H, Medeiros R, Seabra V. SLC19A1, SLC46A1 and SLCO1B1 polymorphisms as predictors of methotrexate-related toxicity in Portuguese rheumatoid arthritis patients. Toxicol Sci 2014;142:196-209.

88. Lima A, Bernardes M, Azevedo R, Seabra V, Medeiros R. Moving toward personalized medicine in rheumatoid arthritis: SNPs in methotrexate intracellular pathways are associated with methotrexate therapeutic outcome. Pharmacogenomics 2016;17:1649-1674.

89. Lima A, Seabra V, Bernardes M, Azevedo R, Sousa H, Medeiros R. Role of key TYMS polymorphisms on methotrexate therapeutic outcome in portuguese rheumatoid arthritis patients. PLoS One 2014;9:e108165.

90. Lu LJ, Bao CD, Dai M, Teng JL, Fan W, Du F, Yang NP, et al. Multicenter, randomized, double-blind, controlled trial of treatment of active rheumatoid arthritis with T-614 compared with methotrexate. Arthritis Rheum 2009;61:979-987.

91. Lv S, Fan H, Li J, Yang H, Huang J, Shu X, Zhang L, et al. Genetic Polymorphisms of TYMS, MTHFR, ATIC, MTR, and MTRR Are Related to the Outcome of Methotrexate Therapy for Rheumatoid Arthritis in a Chinese Population. Rheumatol Int 2018;9:1390.

92. Maini RN, Taylor PC, Szechinski J, Pavelka K, Bröll J, Balint G, Emery P, et al. Double-blind randomized controlled clinical trial of the interleukin-6 receptor antagonist, tocilizumab, in European patients with rheumatoid arthritis who had an incomplete response to methotrexate. Arthritis Rheum 2006;54:2817-2829.

93. McKendry RJ, Cyr M. Toxicity of methotrexate compared with azathioprine in the treatment of rheumatoid arthritis. A case-control study of 131 patients. Arch Intern Med 1989;149:685-689.

94. Mease P, Strand V, Shalamberidze L, Dimic A, Raskina T, Xu LA, Liu Y, et al. A phase II, double-blind, randomised, placebo-controlled study of BMS945429 (ALD518) in patients with rheumatoid arthritis with an inadequate response to methotrexate. Ann Rheum Dis 2012;71:1183-1189.

95. Mena JP, Salazar-Paramo M, Gonzalez-Lopez L, Gamez-Nava JI, Sandoval-Ramirez L, Sanchez JD, Figuera LE, et al. Polymorphisms C677T and A1298C in the MTHFR gene in Mexican patients with rheumatoid arthritis treated with methotrexate: implication with elevation of transaminases. Pharmacogenomics J 2011;11:287-291.

96. Migita K, Akeda Y, Akazawa M, Tohma S, Hirano F, Ideguchi H, Matsumura R, et al. Opsonic and Antibody Responses to Pneumococcal Polysaccharide in Rheumatoid Arthritis Patients Receiving Golimumab Plus Methotrexate. Medicine (Baltimore) 2015;94:e2184.

97. Migliore A, Bizzi E, Massafra U, Vacca F, Martin Martin LS, Ferlito C, Podestà E, et al. Can Cyclosporine-A associated to methotrexate maintain remission induced by anti-TNF agents in rheumatoid arthritis patients? (Cynar pilot study). Int J Immunopathol Pharmacol 2010;23:783-790.

98. Mor A, Bingham CO, 3rd, Kishimoto M, Izmirly PM, Greenberg JD, Reddy S, Rosenthal PB. Methotrexate combined with isoniazid treatment for latent tuberculosis is well tolerated in patients with rheumatoid arthritis: experience from an urban arthritis clinic. Ann Rheum Dis 2008;67:462-465.

99. Morgan SL, Baggott JE, Refsum H, Ueland PM. Homocysteine levels in patients with rheumatoid arthritis treated with low-dose methotrexate. Clin Pharmacol Ther 1991;50:547-556.

100. Mori S, Arima N, Ito M, Fujiyama S, Kamo Y, Ueki Y. Non-alcoholic steatohepatitis-like pattern in liver biopsy of rheumatoid arthritis patients with persistent transaminitis during low-dose methotrexate treatment. PLoS One 2018;13:e0203084.

101. Mori S, Arima N, Ito M, Ueki Y, Abe Y, Aoyagi K, Fujiyama S. Incidence, predictive factors and severity of methotrexate-related liver injury in rheumatoid arthritis: a longitudinal cohort study. Rheumatol Adv Pract 2020;4:rkaa020.

102. Muralidharan N, Mariaselvam CM, Jain VK, Gulati R, Negi VS. ATIC 347C>G gene polymorphism may be associated with methotrexate-induced adverse events in south Indian Tamil rheumatoid arthritis. Pharmacogenomics 2016;17:241-248.

103. Muralidharan N, Mariaselvam CM, Mithun CB, Negi VS. Reduced folate carrier-1 80G > A gene polymorphism is not associated with methotrexate treatment response in South Indian Tamils with rheumatoid arthritis. Clin Rheumatol 2016;35:879-885.

104. Muralidharan N, Misra DP, Jain VK, Negi VS. Effect of thymidylate synthase (TYMS) gene polymorphisms with methotrexate treatment outcome in south Indian Tamil patients with rheumatoid arthritis. Clin Rheumatol 2017;36:1253-1259.

105. Nagaoka S, Katayama K, Kasama T, Sato M, Ohno S, Amasaki Y, Kataoka H, et al. Weekly split-dose regimen for oral methotrexate reduced polyglutamation in red blood cells in patients with rheumatoid arthritis compared with single-dose regimen: Results from a multicentered randomized control trial. 2020;23:1328-1336.

106. Nikiphorou E, Negoescu A, Fitzpatrick JD, Goudie CT, Badcock A, Ostor AJ, Malaviya AP. Indispensable or intolerable? Methotrexate in patients with rheumatoid and psoriatic arthritis: a retrospective review of discontinuation rates from a large UK cohort. Clin Rheumatol 2014;33:609-614.

107. Nishimoto N, Miyasaka N, Yamamoto K, Kawai S, Takeuchi T, Azuma J, Kishimoto T. Study of active controlled tocilizumab monotherapy for rheumatoid arthritis patients with an inadequate response to methotrexate (SATORI): significant reduction in disease activity and serum vascular endothelial growth factor by IL-6 receptor inhibition therapy. Mod Rheumatol 2009;19:12-19.

108. O'Dell JR, Elliott JR, Mallek JA, Mikuls TR, Weaver CA, Glickstein S, Blakely KM, et al. Treatment of early seropositive rheumatoid arthritis: doxycycline plus methotrexate versus methotrexate alone. Arthritis Rheum 2006;54:621-627.

109. O'Dell JR, Haire CE, Erikson N, Drymalski W, Palmer W, Eckhoff PJ, Garwood V, et al. Treatment of rheumatoid arthritis with methotrexate alone, sulfasalazine and hydroxychloroquine, or a combination of all three medications. N Engl J Med 1996;334:1287-1291.

110. Onishi A, Kamitsuji S, Nishida M, Uemura Y, Takahashi M, Saito T, Yoshida Y, et al. Genetic and clinical prediction models for the efficacy and hepatotoxicity of methotrexate in patients with rheumatoid arthritis: a multicenter cohort study. Pharmacogenomics J 2020;20:433-442.

111. Pandya S, Aggarwal A, Misra R. Methotrexate twice weekly vs once weekly in rheumatoid arthritis: a pilot double-blind, controlled study. Rheumatol Int 2002;22:1-4.

112. Park SH, Choe JY, Kim SK. Assessment of liver fibrosis by transient elastography in rheumatoid arthritis patients treated with methotrexate. Joint Bone Spine 2010;77:588-592.

113. Plaza-Plaza JC, Aguilera M, Cañadas-Garre M, Chemello C, González-Utrilla A, Faus Dader MJ, Calleja MA. Pharmacogenetic polymorphisms contributing to toxicity induced by methotrexate in the southern Spanish population with rheumatoid arthritis. Omics 2012;16:589-595.

114. Ranganathan P, Culverhouse R, Marsh S, Mody A, Scott-Horton TJ, Brasington R, Joseph A, et al. Methotrexate (MTX) pathway gene polymorphisms and their effects on MTX toxicity in Caucasian and African American patients with rheumatoid arthritis. J Rheumatol 2008;35:572-579.

115. Rau R, Herborn G, Menninger H, Blechschmidt J. Comparison of intramuscular methotrexate and gold sodium thiomalate in the treatment of early erosive rheumatoid arthritis: 12 month data of a double-blind parallel study of 174 patients. Br J Rheumatol 1997;36:345-352.

116. Richard S, Guerret S, Gerard F, Tebib JG, Vignon E. Hepatic fibrosis in rheumatoid arthritis patients treated with methotrexate: application of a new semi-quantitative scoring system. Rheumatology (Oxford) 2000;39:50-54.

117. Robinson MF, Damjanov N, Stamenkovic B, Radunovic G, Kivitz A, Cox L, Manukyan Z, et al. Efficacy and Safety of PF-06651600 (Ritlecitinib), a Novel JAK3/TEC Inhibitor, in Patients With Moderate-to-Severe Rheumatoid Arthritis and an Inadequate Response to Methotrexate. Arthritis Rheumatol 2020;72:1621-1631.

118. Ros S, Juanola X, Condom E, Cañas C, Riera J, Guardiola J, Del Blanco J, et al. Light and electron microscopic analysis of liver biopsy samples from rheumatoid arthritis patients receiving long-term methotrexate therapy. Scand J Rheumatol 2002;31:330-336.

119. Sakthiswary R, Chan GY, Koh ET, Leong KP. Methotrexate-associated nonalcoholic fatty liver disease with transaminitis in rheumatoid arthritis. 2014;2014:823763.

120. Salesi M, Farajzadegan Z. Efficacy of vitamin D in patients with active rheumatoid arthritis receiving methotrexate therapy. Rheumatol Int 2012;32:2129-2133.

121. Schnabel A, Herlyn K, Burchardi C, Reinhold-Keller E, Gross WL. Long-term tolerability of methotrexate at doses exceeding 15 mg per week in rheumatoid arthritis. Rheumatol Int 1996;15:195-200.

122. Schnabel A, Reinhold-Keller E, Willmann V, Gross WL. Tolerability of methotrexate starting with 15 or 25 mg/week for rheumatoid arthritis. Rheumatol Int 1994;14:33-38.

123. Shergy WJ, Polisson RP, Caldwell DS, Rice JR, Pisetsky DS, Allen NB. Methotrexate-associated hepatotoxicity: retrospective analysis of 210 patients with rheumatoid arthritis. Am J Med 1988;85:771-774.

124. Silverman E, Mouy R, Spiegel L, Jung LK, Saurenmann RK, Lahdenne P, Horneff G, et al. Leflunomide or methotrexate for juvenile rheumatoid arthritis. N Engl J Med 2005;352:1655-1666.

125. Sotoudehmanesh R, Anvari B, Akhlaghi M, Shahraeeni S, Kolahdoozan S. Methotrexate hepatotoxicity in patients with rheumatoid arthritis. Middle East J Dig Dis 2010;2:104-109.

126. Soukup T, Dosedel M, Pavek P, Nekvindova J, Barvik I, Bubancova I, Bradna P, et al. The impact of C677T and A1298C MTHFR polymorphisms on methotrexate therapeutic response in East Bohemian region rheumatoid arthritis patients. Rheumatol Int 2015;35:1149-1161.

127. Stamp LK, OʼDonnell JL, Frampton C, Drake J, Zhang M, Barclay M, Chapman PT. A Pilot Randomized Controlled Double-Blind Trial of High- Versus Low-Dose Weekly Folic Acid in People With Rheumatoid Arthritis Receiving Methotrexate. J Clin Rheumatol 2019;25:284-287.

128. Strand V, Cohen S, Schiff M, Weaver A, Fleischmann R, Cannon G, Fox R, et al. Treatment of active rheumatoid arthritis with leflunomide compared with placebo and methotrexate. Leflunomide Rheumatoid Arthritis Investigators Group. Arch Intern Med 1999;159:2542-2550.

129. Sugiyama E, Moya P, Salazar J, Arranz MJ, Díaz-Torné C, del Río E, Casademont J, et al. Methotrexate pharmacokinetic genetic variants are associated with outcome in rheumatoid arthritis patients. Rheumatol Adv Pract 2016;17:25-29.

130. Sundbaum JK, Baecklund E, Eriksson N, Hallberg P, Kohnke H, Wadelius M. MTHFR, TYMS and SLCO1B1 polymorphisms and adverse liver effects of methotrexate in rheumatoid arthritis. 2020;21:337-346.

131. Suzuki Y, Uehara R, Tajima C, Noguchi A, Ide M, Ichikawa Y, Mizushima Y. Elevation of serum hepatic aminotransferases during treatment of rheumatoid arthritis with low-dose methotrexate. Risk factors and response to folic acid. Scand J Rheumatol 1999;28:273-281.

132. Świerkot J, Batko B, Wiland P, Jędrzejewski M, Stajszczyk M. Methotrexate treatment for rheumatoid arthritis in Poland: Retrospective analysis of patients in routine clinical practice. Reumatologia 2018;56:3-9.

133. Świerkot J, Ślęzak R, Karpiński P, Pawłowska J, Noga L, Szechiński J, Wiland P. Associations between single-nucleotide polymorphisms of RFC-1, GGH, MTHFR , TYMS, and TCII genes and the efficacy and toxicity of methotrexate treatment in patients with rheumatoid arthritis. Pol Arch Med Wewn 2015;125:152-161.

134. Takahashi C, Kaneko Y, Okano Y, Taguchi H, Oshima H, Izumi K, Yamaoka K, et al. Association of erythrocyte methotrexate-polyglutamate levels with the efficacy and hepatotoxicity of methotrexate in patients with rheumatoid arthritis: a 76-week prospective study. RMD Open 2017;3:e000363.

135. Takatori R, Takahashi KA, Tokunaga D, Hojo T, Fujioka M, Asano T, Hirata T, et al. ABCB1 C3435T polymorphism influences methotrexate sensitivity in rheumatoid arthritis patients. Clin Exp Rheumatol 2006;24:546-554.

136. Takeuchi T, Miyasaka N, Zang C, Alvarez D, Fletcher T, Wajdula J, Yuasa H, et al. A phase 3 randomized, double-blind, multicenter comparative study evaluating the effect of etanercept versus methotrexate on radiographic outcomes, disease activity, and safety in Japanese subjects with active rheumatoid arthritis. Mod Rheumatol 2013;23:623-633.

137. Tanaka Y, Suzuki M, Nakamura H, Toyoizumi S, Zwillich SH. Phase II study of tofacitinib (CP-690,550) combined with methotrexate in patients with rheumatoid arthritis and an inadequate response to methotrexate. Ann Rheum Dis 2011;63:1150-1158.

138. Taniguchi A, Urano W, Tanaka E, Furihata S, Kamitsuji S, Inoue E, Yamanaka M, et al. Validation of the associations between single nucleotide polymorphisms or haplotypes and responses to disease-modifying antirheumatic drugs in patients with rheumatoid arthritis: a proposal for prospective pharmacogenomic study in clinical practice. Pharmacogenet Genomics 2007;17:383-390.

139. Taşbaş O, Borman P, Gürhan Karabulut H, Tükün A, Yorgancıoğlu R. The Frequency of A1298C and C677T Polymorphisms of the Methylentetrahydrofolate Gene in Turkish Patients with Rheumatoid Arthritis: Relationship with Methotrexate Toxicity. Open Rheumatol J 2011;5:30-35.

140. Tilling L, Townsend S, David J. Methotrexate and hepatic toxicity in rheumatoid arthritis and psoriatic arthritis. Clin Drug Investig 2006;26:55-62.

141. Tishler M, Caspi D, Rosenbach TO, Fishel B, Wigler I, Segal R, Gazit E, et al. Methotrexate in rheumatoid arthritis: a prospective study in Israeli patients with immunogenetic correlations. Ann Rheum Dis 1988;47:654-659.

142. Urano W, Taniguchi A, Yamanaka H, Tanaka E, Nakajima H, Matsuda Y, Akama H, et al. Polymorphisms in the methylenetetrahydrofolate reductase gene were associated with both the efficacy and the toxicity of methotrexate used for the treatment of rheumatoid arthritis, as evidenced by single locus and haplotype analyses. Pharmacogenetics 2002;12:183-190.

143. van der Heijde D, Tanaka Y, Fleischmann R, Keystone E, Kremer J, Zerbini C, Cardiel MH, et al. Tofacitinib (CP-690,550) in patients with rheumatoid arthritis receiving methotrexate: twelve-month data from a twenty-four-month phase III randomized radiographic study. Arthritis Rheum 2013;65:559-570.

144. van Dongen H, van Aken J, Lard LR, Visser K, Ronday HK, Hulsmans HM, Speyer I, et al. Efficacy of methotrexate treatment in patients with probable rheumatoid arthritis: a double-blind, randomized, placebo-controlled trial. Arthritis Rheum 2007;56:1424-1432.

145. van Ede AE, Laan RF, Blom HJ, Boers GH, Haagsma CJ, Thomas CM, De Boo TM, et al. Homocysteine and folate status in methotrexate-treated patients with rheumatoid arthritis. Rheumatology (Oxford) 2002;41:658-665.

146. van Ede AE, Laan RF, Blom HJ, Huizinga TW, Haagsma CJ, Giesendorf BA, de Boo TM, et al. The C677T mutation in the methylenetetrahydrofolate reductase gene: a genetic risk factor for methotrexate-related elevation of liver enzymes in rheumatoid arthritis patients. Arthritis Rheum 2001;44:2525-2530.

147. van Ede AE, Laan RF, De Abreu RA, Stegeman AB, van de Putte LB. Purine enzymes in patients with rheumatoid arthritis treated with methotrexate. Ann Rheum Dis 2002;61:1060-1064.

148. van Ede AE, Laan RF, Rood MJ, Huizinga TW, van de Laar MA, van Denderen CJ, Westgeest TA, et al. Effect of folic or folinic acid supplementation on the toxicity and efficacy of methotrexate in rheumatoid arthritis: a forty-eight week, multicenter, randomized, double-blind, placebo-controlled study. Arthritis Rheum 2001;44:1515-1524.

149. Vejnović D, Milić V, Popović B, Damnjanović T, Maksimović N, Bunjevački V, Krajinović M, et al. Association of C35T polymorphism in dihydrofolate reductase gene with toxicity of methotrexate in rheumatoid arthritis patients. Expert Opin Drug Metab Toxicol 2019;15:253-257.

150. Verstappen SM, Bakker MF, Heurkens AH, van der Veen MJ, Kruize AA, Geurts MA, Bijlsma JW, et al. Adverse events and factors associated with toxicity in patients with early rheumatoid arthritis treated with methotrexate tight control therapy: the CAMERA study. Ann Rheum Dis 2010;69:1044-1048.

151. Wallace CA, Bleyer WA, Sherry DD, Salmonson KL, Wedgwood RJ. Toxicity and serum levels of methotrexate in children with juvenile rheumatoid arthritis. Arthritis Rheum 1989;32:677-681.

152. Wei W, Zhang LL, Xu JH, Xiao F, Bao CD, Ni LQ, Li XF, et al. A multicenter, double-blind, randomized, controlled phase III clinical trial of chicken type II collagen in rheumatoid arthritis. Arthritis Res Ther 2009;11:R180.

153. Weinblatt ME, Genovese MC, Ho M, Hollis S, Rosiak-Jedrychowicz K, Kavanaugh A, Millson DS, et al. Effects of fostamatinib, an oral spleen tyrosine kinase inhibitor, in rheumatoid arthritis patients with an inadequate response to methotrexate: results from a phase III, multicenter, randomized, double-blind, placebo-controlled, parallel-group study. Arthritis Rheumatol 2014;66:3255-3264.

154. Weinblatt ME, Kaplan H, Germain BF, Merriman RC, Solomon SD, Wall B, Anderson L, et al. Low-dose methotrexate compared with auranofin in adult rheumatoid arthritis. A thirty-six-week, double-blind trial. Arthritis Rheum 1990;33:330-338.

155. Weinblatt ME, Keystone EC, Furst DE, Moreland LW, Weisman MH, Birbara CA, Teoh LA, et al. Adalimumab, a fully human anti-tumor necrosis factor alpha monoclonal antibody, for the treatment of rheumatoid arthritis in patients taking concomitant methotrexate: the ARMADA trial. Arthritis Rheum 2003;48:35-45.

156. Weinblatt ME, Peloso PM, Chen K, Othman AA, Li Y, Mansikka HT, Khatri A, et al. Risk of liver disease in patients with psoriasis, psoriatic arthritis, and rheumatoid arthritis receiving methotrexate: a population-based study. Arthritis Rheumatol 2021.

157. Weinblatt ME, Weissman BN, Holdsworth DE, Fraser PA, Maier AL, Falchuk KR, Coblyn JS. Long-term prospective study of methotrexate in the treatment of rheumatoid arthritis. 84-month update. Arthritis Rheum 1992;35:129-137.

158. Weinstein A, Marlowe S, Korn J, Farouhar F. Low-dose methotrexate treatment of rheumatoid arthritis. Long-term observations. Am J Med 1985;79:331-337.

159. Weisman MH, Furst DE, Park GS, Kremer JM, Smith KM, Wallace DJ, Caldwell JR, et al. Risk genotypes in folate-dependent enzymes and their association with methotrexate-related side effects in rheumatoid arthritis. Arthritis Rheum 2006;54:607-612.

160. Weisman MH, Moreland LW, Furst DE, Weinblatt ME, Keystone EC, Paulus HE, Teoh LS, et al. Efficacy, pharmacokinetic, and safety assessment of adalimumab, a fully human anti-tumor necrosis factor-alpha monoclonal antibody, in adults with rheumatoid arthritis receiving concomitant methotrexate: a pilot study. Clin Ther 2003;25:1700-1721.

161. Wessels JA, de Vries-Bouwstra JK, Heijmans BT, Slagboom PE, Goekoop-Ruiterman YP, Allaart CF, Kerstens PJ, et al. Efficacy and toxicity of methotrexate in early rheumatoid arthritis are associated with single-nucleotide polymorphisms in genes coding for folate pathway enzymes. Arthritis Rheum 2006;54:1087-1095.

162. Wessels JA, Kooloos WM, De Jonge R, De Vries-Bouwstra JK, Allaart CF, Linssen A, Collee G, et al. Relationship between genetic variants in the adenosine pathway and outcome of methotrexate treatment in patients with recent-onset rheumatoid arthritis. Arthritis Rheum 2006;54:2830-2839.

163. Westhovens R, Taylor PC, Alten R, Pavlova D, Enríquez-Sosa F, Mazur M, Greenwald M, et al. Filgotinib (GLPG0634/GS-6034), an oral JAK1 selective inhibitor, is effective in combination with methotrexate (MTX) in patients with active rheumatoid arthritis and insufficient response to MTX: results from a randomised, dose-finding study (DARWIN 1). Ann Rheum Dis 2017;76:998-1008.

164. Williams HJ, Ward JR, Reading JC, Brooks RH, Clegg DO, Skosey JL, Weisman MH, et al. Comparison of auranofin, methotrexate, and the combination of both in the treatment of rheumatoid arthritis. A controlled clinical trial. Arthritis Rheum 1992;35:259-269.

165. Williams HJ, Willkens RF, Samuelson CO, Jr., Alarcón GS, Guttadauria M, Yarboro C, Polisson RP, et al. Comparison of low-dose oral pulse methotrexate and placebo in the treatment of rheumatoid arthritis. A controlled clinical trial. Arthritis Rheum 1985;28:721-730.

166. Willkens RF, Leonard PA, Clegg DO, Tolman KG, Ward JR, Marks CR, Greene ML, et al. Liver histology in patients receiving low dose pulse methotrexate for the treatment of rheumatoid arthritis. Ann Rheum Dis 1990;49:591-593.

167. Willkens RF, Urowitz MB, Stablein DM, McKendry RJ, Jr., Berger RG, Box JH, Fiechtner JJ, et al. Comparison of azathioprine, methotrexate, and the combination of both in the treatment of rheumatoid arthritis. A controlled clinical trial. Arthritis Rheum 1992;35:849-856.

168. Xiang N, Li XM, Zhang MJ, Zhao DB, Zhu P, Zuo XX, Yang M, et al. Total glucosides of paeony can reduce the hepatotoxicity caused by Methotrexate and Leflunomide combination treatment of active rheumatoid arthritis. Int Immunopharmacol 2015;28:802-807.

169. Yamamoto K, Takeuchi T, Yamanaka H, Ishiguro N, Tanaka Y, Eguchi K, Watanabe A, et al. Efficacy and safety of certolizumab pegol plus methotrexate in Japanese rheumatoid arthritis patients with an inadequate response to methotrexate: the J-RAPID randomized, placebo-controlled trial. Mod Rheumatol 2014;24:715-724.

170. Yazici Y, Erkan D, Harrison MJ, Nikolov NP, Paget SA. Methotrexate use in rheumatoid arthritis is associated with few clinically significant liver function test abnormalities. Clin Exp Rheumatol 2005;23:517-520.

171. Yonemoto Y, Okamura K, Takeuchi K, Ayabe K, Kaneko T, Matsushita M, Tamura Y, et al. Comparison of golimumab 100-mg monotherapy to golimumab 50 mg plus methotrexate in patients with rheumatoid arthritis: Results from a multicenter, cohort study. Mod Rheumatol 2016;26:24-28.

172. Zhang LL, Wei W, Xiao F, Xu JH, Bao CD, Ni LQ, Li XF. A randomized, double-blind, multicenter, controlled clinical trial of chicken type II collagen in patients with rheumatoid arthritis. Arthritis Rheum 2008;59:905-910.

**Included articles of psoriasis/psoriatic arthritis:**

1. Abidi A, Rizvi DA, Saxena K, Chaudhary S, Ahmad A. The evaluation of efficacy and safety of methotrexate and pioglitazone in psoriasis patients: A randomized, open-labeled, active-controlled clinical trial. Indian J Pharmacol 2020;52:16-22.

2. Aithal GP, Haugk B, Das S, Card T, Burt AD, Record CO. Monitoring methotrexate-induced hepatic fibrosis in patients with psoriasis: are serial liver biopsies justified? Aliment Pharmacol Ther 2004;19:391-399.

3. Al-Hamamy HR, Al-Mashhadani SA, Mustafa IN. Comparative study of the effect of narrowband ultraviolet B phototherapy plus methotrexate vs. narrowband ultraviolet B alone and methotrexate alone in the treatment of plaque-type psoriasis. Int J Dermatol 2014;53:1531-1535.

4. Amital H, Arnson Y, Chodick G, Shalev V. Hepatotoxicity rates do not differ in patients with rheumatoid arthritis and psoriasis treated with methotrexate. Rheumatology (Oxford) 2009;48:1107-1110.

5. Appani SK, Devarasetti PK, Irlapati RVP, Rajasekhar L. Methotrexate achieves major cDAPSA response, and improvement in dactylitis and functional status in psoriatic arthritis. Rheumatology (Oxford) 2019;58:869-873.

6. Ashton RE, Millward-Sadler GH, White JE. Complications in methotrexate treatment of psoriasis with particular reference to liver fibrosis. J Invest Dermatol 1982;79:229-232.

7. Attwa EM, Elkot RA. Subcutaneous methotrexate versus oral form for the treatment and prophylaxis of chronic plaque psoriasis. 2019;32:e13051.

8. Aydin F, Canturk T, Senturk N, Turanli AY. Methotrexate and ciclosporin combination for the treatment of severe psoriasis. Clin Exp Dermatol 2006;31:520-524.

9. Barker J, Hoffmann M, Wozel G, Ortonne JP, Zheng H, van Hoogstraten H, Reich K. Efficacy and safety of infliximab vs. methotrexate in patients with moderate-to-severe plaque psoriasis: results of an open-label, active-controlled, randomized trial (RESTORE1). Br J Dermatol 2011;165:1109-1117.

10. Berends MA, Snoek J, de Jong EM, van de Kerkhof PC, van Oijen MG, van Krieken JH, Drenth JP. Liver injury in long-term methotrexate treatment in psoriasis is relatively infrequent. Aliment Pharmacol Ther 2006;24:805-811.

11. Berends MA, Snoek J, de Jong EM, Van Krieken JH, de Knegt RJ, van Oijen MG, van de Kerkhof PC, et al. Biochemical and biophysical assessment of MTX-induced liver fibrosis in psoriasis patients: Fibrotest predicts the presence and Fibroscan predicts the absence of significant liver fibrosis. Liver Int 2007;27:639-645.

12. Bronckers I, Paller AS, West DP, Lara-Corrales I, Tollefson MM, Tom WL, Hogeling M, et al. A Comparison of Psoriasis Severity in Pediatric Patients Treated With Methotrexate vs Biologic Agents. JAMA Dermatol 2020;156:384-392.

13. Cabello Zurita C, Grau Pérez M, Hernández Fernández CP, González Quesada A, Valerón Almazán P, Vilar Alejo J, Carretero Hernández G. Effectiveness and safety of Methotrexate in psoriasis: an eight-year experience with 218 patients. Br J Dermatol 2017;28:401-405.

14. Chandran V, Siannis F, Rahman P, Pellett FJ, Farewell VT, Gladman DD. Folate pathway enzyme gene polymorphisms and the efficacy and toxicity of methotrexate in psoriatic arthritis. J Rheumatol 2010;37:1508-1512.

15. Chládek J, Simková M, Vanecková J, Hroch M, Chládkova J, Martínková J, Vávrová J, et al. The effect of folic acid supplementation on the pharmacokinetics and pharmacodynamics of oral methotrexate during the remission-induction period of treatment for moderate-to-severe plaque psoriasis. Eur J Clin Pharmacol 2008;64:347-355.

16. Chládek J, Simková M, Vanecková J, Hroch M, Vávrová J, Hůlek P. Assessment of methotrexate hepatotoxicity in psoriasis patients: a prospective evaluation of four serum fibrosis markers. J Eur Acad Dermatol Venereol 2013;27:1007-1014.

17. Choi CW, Kim BR, Ohn J, Youn SW. The Advantage of Cyclosporine A and Methotrexate Rotational Therapy in Long-Term Systemic Treatment for Chronic Plaque Psoriasis in a Real World Practice. Ann Dermatol 2017;29:55-60.

18. Coates LC, Helliwell PS. Methotrexate Efficacy in the Tight Control in Psoriatic Arthritis Study. J Rheumatol 2016;43:356-361.

19. de Jong HMY, van Mens LJJ, Nurmohamed MT, Kok MR, van Kuijk AWR, Baeten DLP, van de Sande MGH. Sustained remission with methotrexate monotherapy after 22-week induction treatment with TNF-alpha inhibitor and methotrexate in early psoriatic arthritis: an open-label extension of a randomized placebo-controlled trial. Arthritis Res Ther 2019;21:208.

20. Dogra S, Krishna V, Kanwar AJ. Efficacy and safety of systemic methotrexate in two fixed doses of 10 mg or 25 mg orally once weekly in adult patients with severe plaque-type psoriasis: a prospective, randomized, double-blind, dose-ranging study. Clin Exp Dermatol 2012;37:729-734.

21. Drach M, Papageorgiou K, Maul JT, Djamei V, Yawalkar N, Häusermann P, Anzengruber F, et al. Effectiveness of methotrexate in moderate to severe psoriasis patients: real-world registry data from the Swiss Dermatology Network for Targeted Therapies (SDNTT). Arch Dermatol Res 2019;311:753-760.

22. Fallah Arani S, Neumann H, Hop WC, Thio HB. Fumarates vs. methotrexate in moderate to severe chronic plaque psoriasis: a multicentre prospective randomized controlled clinical trial. Br J Dermatol 2011;164:855-861.

23. Flytström I, Stenberg B, Svensson A, Bergbrant IM. Methotrexate vs. ciclosporin in psoriasis: effectiveness, quality of life and safety. A randomized controlled trial. Br J Dermatol 2008;158:116-121.

24. Gelfand JM, Wan J, Zhang H, Shin DB, Ogdie A, Syed MN, Egeberg A. Risk of liver disease in patients with psoriasis, psoriatic arthritis, and rheumatoid arthritis receiving methotrexate: a population-based study. J Am Acad Dermatol 2021.

25. Geronemus RG, Auerbach R, Tobias H. Liver biopsies upsilon liver scans in methotrexate-treated patients with psoriasis. Arch Dermatol 1982;118:649-651.

26. Ghariani N, Sriha B, Belajouza C, Denguezli M, Nouira R, Tsakok T. Subcutaneous methotrexate in patients with moderate-to-severe psoriasis: a critical appraisal. Dermatol Ther (Heidelb) 2018;179:50-53.

27. Gisondi P, Bellinato F. Methotrexate vs secukinumab safety in psoriasis patients with metabolic syndrome. 2020;33:e14281.

28. Gordon KB, Betts KA, Sundaram M, Signorovitch JE, Li J, Xie M, Wu EQ, et al. Poor early response to methotrexate portends inadequate long-term outcomes in patients with moderate-to-severe psoriasis: Evidence from 2 phase 3 clinical trials. J Am Acad Dermatol 2017;77:1030-1037.

29. Gottlieb AB, Langley RG, Strober BE, Papp KA, Klekotka P, Creamer K, Thompson EH, et al. A randomized, double-blind, placebo-controlled study to evaluate the addition of methotrexate to etanercept in patients with moderate to severe plaque psoriasis. Br J Dermatol 2012;167:649-657.

30. Gupta R, Gupta S. Methotrexate-betamethasone weekly oral pulse in psoriasis. J Dermatolog Treat 2007;18:291-294.

31. Gupta SK, Singh KK, Lalit M. Comparative therapeutic evaluation of different topicals and narrow band ultraviolet B therapy combined with systemic methotrexate in the treatment of palmoplantar psoriasis. Indian J Dermatol 2011;56:165-170.

32. Haider S, Wahid Z, Najam Us S, Riaz F. Efficacy of Methotrexate in patients with plaque type psoriasis. Pak J Med Sci 2014;30:1050-1053.

33. Harrison PV, Orrell DH, James R, Peat M, Stones RN. Short-term methotrexate administration by low-dose infusion--does it influence clearance of psoriasis? Clin Exp Dermatol 1989;14:291-294.

34. Haustein UF, Rytter M. Methotrexate in psoriasis: 26 years' experience with low-dose long-term treatment. J Eur Acad Dermatol Venereol 2000;14:382-388.

35. Heiberg MS, Kaufmann C, Rødevand E, Mikkelsen K, Koldingsnes W, Mowinckel P, Kvien TK. The comparative effectiveness of anti-TNF therapy and methotrexate in patients with psoriatic arthritis: 6 month results from a longitudinal, observational, multicentre study. Ann Rheum Dis 2007;66:1038-1042.

36. Heydendael VM, Spuls PI, Opmeer BC, de Borgie CA, Reitsma JB, Goldschmidt WF, Bossuyt PM, et al. Methotrexate versus cyclosporine in moderate-to-severe chronic plaque psoriasis. N Engl J Med 2003;349:658-665.

37. Inzinger M, Weger W, Heschl B, Salmhofer W, Quehenberger F, Wolf P. Methotrexate vs. fumaric acid esters in moderate-to-severe chronic plaque psoriasis: data registry report on the efficacy under daily life conditions. J Eur Acad Dermatol Venereol 2013;27:861-866.

38. Jacobs ME, Pouw JN, Welsing P, Radstake T, Leijten EFA. First-line csDMARD monotherapy drug retention in psoriatic arthritis: methotrexate outperforms sulfasalazine. Rheumatology (Oxford) 2021;60:780-784.

39. Janagond AB, Kanwar AJ, Handa S. Efficacy and safety of systemic methotrexate vs. acitretin in psoriasis patients with significant palmoplantar involvement: a prospective, randomized study. J Eur Acad Dermatol Venereol 2013;27:e384-389.

40. Kaur I, Dogra S, De D, Kanwar AJ. Systemic methotrexate treatment in childhood psoriasis: further experience in 24 children from India. Pediatr Dermatol 2008;25:184-188.

41. Khan S, Subedi D, Chowdhury MM. Use of amino terminal type III procollagen peptide (P3NP) assay in methotrexate therapy for psoriasis. Postgrad Med J 2006;82:353-354.

42. Khandpur S, Yadav D, Jangid B, Kumar A, Shalimar, Devasenathipathy K, Sharma R, et al. Ultrasound liver elastography for the detection of liver fibrosis in patients with psoriasis and reactive arthritis on long-term methotrexate therapy: A cross-sectional study. Indian J Dermatol Venereol Leprol 2020;86:508-514.

43. Kilic S, Emre S, Metin A, Isikoglu S, Erel O. Effect of the systemic use of methotrexate on the oxidative stress and paraoxonase enzyme in psoriasis patients. Arch Dermatol Res 2013;305:495-500.

44. Kingsley GH, Kowalczyk A, Taylor H, Ibrahim F, Packham JC, McHugh NJ, Mulherin DM, et al. A randomized placebo-controlled trial of methotrexate in psoriatic arthritis. Rheumatology (Oxford) 2012;51:1368-1377.

45. Kumar B, Saraswat A, Kaur I. Short-term methotrexate therapy in psoriasis: a study of 197 patients. Int J Dermatol 2002;41:444-448.

46. Lajevardi V, Hallaji Z, Daklan S, Abedini R, Goodarzi A, Abdolreza M. The efficacy of methotrexate plus pioglitazone vs. methotrexate alone in the management of patients with plaque-type psoriasis: a single-blinded randomized controlled trial. Int J Dermatol 2015;54:95-101.

47. Lanse SB, Arnold GL, Gowans JD, Kaplan MM. Low incidence of hepatotoxicity associated with long-term, low-dose oral methotrexate in treatment of refractory psoriasis, psoriatic arthritis, and rheumatoid arthritis. An acceptable risk/benefit ratio. Dig Dis Sci 1985;30:104-109.

48. Lie E, van der Heijde D, Uhlig T, Heiberg MS, Koldingsnes W, Rødevand E, Kaufmann C, et al. Effectiveness and retention rates of methotrexate in psoriatic arthritis in comparison with methotrexate-treated patients with rheumatoid arthritis. Ann Rheum Dis 2010;69:671-676.

49. Liu LF, Chen JS, Gu J, Xu JH, Jin HZ, Pang XW, Wang G, et al. Etanercept biosimilar (recombinant human tumor necrosis factor-α receptor II: IgG Fc fusion protein) and methotrexate combination therapy in Chinese patients with moderate-to-severe plaque psoriasis: a multicentre, randomized, double-blind, placebo-controlled trial. 2020;312:437-445.

50. Lynch M, Higgins E, McCormick PA, Kirby B, Nolan N, Rogers S, Lally A, et al. The use of transient elastography and FibroTest for monitoring hepatotoxicity in patients receiving methotrexate for psoriasis. JAMA Dermatol 2014;150:856-862.

51. Mahajan R, Dogra S, Handa S, Razmi TM, Narang T, Rathi S, Dhiman RK, et al. Metabolic syndrome and female gender, but not methotrexate, are the important associations of significant liver fibrosis in patients with moderate-to-severe psoriasis as detected by transient elastography. Dermatol Ther 2020;86:649-655.

52. Malesci D, Tirri R, Buono R, La Montagna G. Leflunomide in psoriatic arthritis: a retrospective study of discontinuation rate in daily clinical practice compared with methotrexate. Clin Exp Rheumatol 2007;25:881-884.

53. Mease PJ, Genovese MC. Phase II Study of ABT-122, a Tumor Necrosis Factor- and Interleukin-17A-Targeted Dual Variable Domain Immunoglobulin, in Patients With Psoriatic Arthritis With an Inadequate Response to Methotrexate. 2018;70:1778-1789.

54. Mease PJ, Gladman DD, Collier DH, Ritchlin CT, Helliwell PS, Liu L, Kricorian G, et al. Etanercept and Methotrexate as Monotherapy or in Combination for Psoriatic Arthritis: Primary Results From a Randomized, Controlled Phase III Trial. Arthritis Rheumatol 2019;71:1112-1124.

55. Mease PJ, Gladman DD, Keystone EC. Alefacept in combination with methotrexate for the treatment of psoriatic arthritis: results of a randomized, double-blind, placebo-controlled study. Arthritis Rheum 2006;54:1638-1645.

56. Morison WL, Momtaz K, Parrish JA, Fitzpatrick TB. Combined methotrexate-PUVA therapy in the treatment of psoriasis. J Am Acad Dermatol 1982;6:46-51.

57. Nikiphorou E, Negoescu A, Fitzpatrick JD, Goudie CT, Badcock A, Östör AJ, Malaviya AP. Indispensable or intolerable? Methotrexate in patients with rheumatoid and psoriatic arthritis: a retrospective review of discontinuation rates from a large UK cohort. Clin Rheumatol 2014;33:609-614.

58. Otero ME, van den Reek JM, Seyger MM, van de Kerkhof PC, Kievit W, de Jong EM. Determinants for drug survival of methotrexate in patients with psoriasis, split according to different reasons for discontinuation: results of the prospective MTX-CAPTURE. Br J Dermatol 2017;177:497-504.

59. Papp K, Thaçi D, Marcoux D, Weibel L, Philipp S, Ghislain PD, Landells I, et al. Efficacy and safety of adalimumab every other week versus methotrexate once weekly in children and adolescents with severe chronic plaque psoriasis: a randomised, double-blind, phase 3 trial. Lancet 2017;390:40-49.

60. Paul BS, Momtaz K, Stern RS, Arndt KA, Parrish JA. Combined methotrexate--ultraviolet B therapy in the treatment of psoriasis. J Am Acad Dermatol 1982;7:758-762.

61. Pongparit K, Chularojanamontri L. Effectiveness of and factors associated with clinical response to methotrexate under daily life conditions in Asian patients with psoriasis: A retrospective cohort study. 2018;45:540-545.

62. Radmanesh M, Rafiei B, Moosavi ZB, Sina N. Weekly vs. daily administration of oral methotrexate (MTX) for generalized plaque psoriasis: a randomized controlled clinical trial. Int J Dermatol 2011;50:1291-1293.

63. Reich K, Augustin M. A 24-week multicentre, randomized, open-label, parallel-group study comparing the efficacy and safety of ixekizumab vs. fumaric acid esters and methotrexate in patients with moderate-to-severe plaque psoriasis naive to systemic treatment. 2020;182:869-879.

64. Reich K, Langley RG, Papp KA, Ortonne JP, Unnebrink K, Kaul M, Valdes JM. A 52-week trial comparing briakinumab with methotrexate in patients with psoriasis. N Engl J Med 2011;365:1586-1596.

65. Richter S, Guerette B, Mease PJ, Thi VB, Minh VN, Ngoc AT, Dang QT, et al. Effectiveness, Safety and Tolerance of Methotrexate in Vietnamese Psoriatic Arthritis Patients. J Rheumatol 2019;7:250-252.

66. Robinson JK, Baughman RD, Auerbach R, Cimis RJ. Methotrexate hepatotoxicity in psoriasis. Consideration of liver biopsies at regular intervals. Arch Dermatol 1980;116:413-415.

67. Rongngern P, Chularojanamontri L, Wongpraparut C, Silpa-Archa N, Chotiyaputta W, Pongpaibul A, Charatcharoenwitthaya P. Diagnostic performance of transient elastography for detection of methotrexate-induced liver injury using Roenigk classification in Asian patients with psoriasis: a retrospective study. Arch Dermatol Res 2017;309:403-408.

68. Salim A, Tan E, Ilchyshyn A, Berth-Jones J. Folic acid supplementation during treatment of psoriasis with methotrexate: a randomized, double-blind, placebo-controlled trial. Br J Dermatol 2006;154:1169-1174.

69. Santos FCF, Montenegro LML, Silva MDC, Souza VMO, Lima Neto RG, Moura L, Magalhães V, et al. Methotrexate efficacy and tolerance in plaque psoriasis. A prospective real-life multicentre study in France. PLoS One 2019;146:106-114.

70. Saurat JH, Guérin A, Yu AP, Latremouille-Viau D, Wu EQ, Gupta SR, Bao Y, et al. High prevalence of potential drug-drug interactions for psoriasis patients prescribed methotrexate or cyclosporine for psoriasis: associated clinical and economic outcomes in real-world practice. Dermatology 2010;220:128-137.

71. Saurat JH, Langley RG, Reich K, Unnebrink K, Sasso EH, Kampman W. Relationship between methotrexate dosing and clinical response in patients with moderate to severe psoriasis: subanalysis of the CHAMPION study. Br J Dermatol 2011;165:399-406.

72. Saurat JH, Stingl G, Dubertret L, Papp K, Langley RG, Ortonne JP, Unnebrink K, et al. Efficacy and safety results from the randomized controlled comparative study of adalimumab vs. methotrexate vs. placebo in patients with psoriasis (CHAMPION). Br J Dermatol 2008;158:558-566.

73. Scarpa R, Peluso R, Atteno M, Manguso F, Spanò A, Iervolino S, Di Minno MN, et al. The effectiveness of a traditional therapeutical approach in early psoriatic arthritis: results of a pilot randomised 6-month trial with methotrexate. Clin Rheumatol 2008;27:823-826.

74. Shah S, Nikam B, Kale M, Jamale V, Chavan D. Safety and efficacy profile of oral cyclosporine vs oral methotrexate vs oral acitretin in palmoplantar psoriasis: A hospital based prospective investigator blind randomized controlled comparative study. Dermatol Ther 2021;34:e14650.

75. Sheane BJ, Thavaneswaran A, Gladman DD, Chandran V. Attainment of Minimal Disease Activity Using Methotrexate in Psoriatic Arthritis. J Rheumatol 2016;43:1718-1723.

76. Shehzad T, Dar NR, Zakria M. Efficacy of concomitant use of PUVA and methotrexate in disease clearance time in plaque type psoriasis. J Pak Med Assoc 2004;54:453-455.

77. Singh SK, Rai T. Relapse in psoriasis with two different tapering regimens of methotrexate: a randomized open-label controlled study. Indian J Dermatol Venereol Leprol 2015;81:144-147.

78. Smolen JS, Sebba A, Ruderman EM, Schulze-Koops H, Sapin C, Gellett AM, Sprabery AT, et al. Efficacy and Safety of Ixekizumab with or Without Methotrexate in Biologic-Naïve Patients with Psoriatic Arthritis: 52-Week Results from SPIRIT-H2H Study. Rheumatol Ther 2020;7:1021-1035.

79. Stolshek BS, Liu LXH, Collier DH, Kricorian G, Merola JF, van Mens LJJ. Achieving remission in psoriatic arthritis by early initiation of TNF inhibition: a double-blind, randomised, placebo-controlled trial of golimumab plus methotrexate versus placebo plus methotrexate. RMD Open 2019;78:610-616.

80. Tamilselvi E, Haripriya D, Hemamalini M, Pushpa G, Swapna S. Association of disease severity with IL-1 levels in methotrexate-treated psoriasis patients. Scand J Immunol 2013;78:545-553.

81. Tilling L, Townsend S, David J. Methotrexate and hepatic toxicity in rheumatoid arthritis and psoriatic arthritis. Clin Drug Investig 2006;26:55-62.

82. van de Kerkhof PC, Hoefnagels WH, van Haelst UJ, Mali JW. Methotrexate maintenance therapy and liver damage in psoriasis. Clin Exp Dermatol 1985;10:194-200.

83. Van Dooren-Greebe RJ, Kuijpers AL, Mulder J, De Boo T, Van de Kerkhof PC. Methotrexate revisited: effects of long-term treatment in psoriasis. Br J Dermatol 1994;130:204-210.

84. Van ED, Diem TP, Thi VB, Xuan THT, Tuan KL, Quynh TN, Thu TV, et al. Successful Psoriasis Treatment Using NB-UVB with Methotrexate: The Vietnamese Experience. Clin Exp Dermatol 2019;7:253-255.

85. van Geel MJ, Oostveen AM, Hoppenreijs EP, Hendriks JC, van de Kerkhof PC, de Jong EM, Seyger MM. Methotrexate in pediatric plaque-type psoriasis: Long-term daily clinical practice results from the Child-CAPTURE registry. J Dermatolog Treat 2015;26:406-412.

86. van Swelm RP, Laarakkers CM, Kooijmans-Otero M, de Jong EM, Masereeuw R, Russel FG. Biomarkers for methotrexate-induced liver injury: urinary protein profiling of psoriasis patients. Toxicol Lett 2013;221:219-224.

87. vanDooren-Greebe RJ, Kuijpers AL, Buijs WC, Kniest PH, Corstens FH, Nagengast FM, de Boo T, et al. The value of dynamic hepatic scintigraphy and serum aminoterminal propeptide of type III procollagen for early detection of methotrexate-induced hepatic damage in psoriasis patients. Br J Dermatol 1996;134:481-487.

88. Warren RB, Mrowietz U, von Kiedrowski R, Niesmann J, Wilsmann-Theis D, Ghoreschi K, Zschocke I, et al. An intensified dosing schedule of subcutaneous methotrexate in patients with moderate to severe plaque-type psoriasis (METOP): a 52 week, multicentre, randomised, double-blind, placebo-controlled, phase 3 trial. Br J Dermatol 2017;389:528-537.

89. Warren RB, Smith RL, Campalani E, Eyre S, Smith CH, Barker JN, Worthington J, et al. Genetic variation in efflux transporters influences outcome to methotrexate therapy in patients with psoriasis. J Invest Dermatol 2008;128:1925-1929.

90. West J, Ogston S, Palmer C, Fleming C, Dawe R, Kumar V, Waterston S, et al. Methotrexate in psoriasis under real-world conditions: long-term efficacy and tolerability. Br J Dermatol 2016;174:1407-1410.

91. Woolf RT, West SL, Arenas-Hernandez M, Hare N, Peters van Ton AM, Lewis CM, Marinaki AM, et al. Methotrexate polyglutamates as a marker of patient compliance and clinical response in psoriasis: a single-centre prospective study. Br J Dermatol 2012;167:165-173.

92. Wysoczańska B, Korman L, Wiland P, Bogunia-Kubik K, Espinoza LR, Zakraoui L, Espinoza CG, et al. Psoriatic arthritis: clinical response and side effects to methotrexate therapy. Pharmgenomics Pers Med 1992;19:872-877.

93. Yan K, Zhang Y, Han L, Huang Q, Zhang Z, Fang X, Zheng Z, et al. Safety and Efficacy of Methotrexate for Chinese Adults With Psoriasis With and Without Psoriatic Arthritis. JAMA Dermatol 2019;155:327-334.

94. Yeo CM, Chong VH, Earnest A, Yang WL. Prevalence and risk factors of methotrexate hepatoxicity in Asian patients with psoriasis. World J Hepatol 2013;5:275-280.

95. Youness ER, Aboel-Fadl DM, Collins P, Rogers S. The efficacy of methotrexate in psoriasis--a review of 40 cases. Artif Cells Nanomed Biotechnol 1992;17:257-260.

96. Yousefzadeh H, Jabbari Azad F, Banihashemi M, Rastin M, Mahmoudi M. Evaluation of psoriasis severity and inflammatory responses under concomitant treatment with methotrexate plus micronutrients for psoriasis vulgaris: a randomized double blind trial. Acta Dermatovenerol Alp Pannonica Adriat 2017;26:3-9.
